# Efficacy and safety of higher dose rifampicin in adults with presumed drug-susceptible tuberculosis: an updated systematic review and meta-analysis

**DOI:** 10.1016/j.eclinm.2024.102857

**Published:** 2024-10-03

**Authors:** Kathryn A. Haigh, Hussein H. Twabi, Linda Boloko, Phiona E. Namale, Vittoria Lutje, Sarah Nevitt, Geraint Davies

**Affiliations:** a Department for Clinical Infection, Microbiology and Immunology, Institute of Infection, Veterinary and Ecological Sciences, University of Liverpool, UK; b Centre for Infectious Diseases Research in Africa, Institute of Infectious Disease and Molecular Medicine, University of Cape Town, South Africa; c Kamuzu University of Health Sciences, Blantyre, Malawi; d Institute of Life Course and Medical Sciences, University of Liverpool, UK; e Division of Infectious Diseases and HIV Medicine, Department of Medicine, Groote Schuur Hospital, Cape Town, South Africa; f Cochrane Infectious Diseases Group, Liverpool School of Tropical Medicine, Liverpool, UK; g Department of Health Data Science, Institute of Population Health, University of Liverpool, UK; h Centre for Reviews and Dissemination, University of York, York, UK

**Keywords:** High dose rifampicin, Optimisation of rifampicin, Tuberculosis, Systematic review, Meta-analysis

## Abstract

**Background:**

Tuberculosis (TB) remains a significant cause of mortality globally, yet first-line treatment has hardly changed for fifty years. The dose of rifampicin, the most important drug in this regimen, has been historically based on pragmatic cost- and risk-benefit considerations. Evidence suggests the current recommended dose (8–12 mg/kg) may not maximise the potential benefits of this drug. We sought to evaluate the efficacy and safety of higher doses of rifampicin in adults with presumed drug-susceptible TB.

**Methods:**

In this systematic review we searched MEDLINE, EMBASE, CENTRAL and Global Health databases for randomised controlled trials up to 31 July 2024 of adults with presumed drug-susceptible TB receiving first-line treatment with an intervention of rifampicin doses higher than currently recommended. Meta-analyses were performed using random effects models where background regimens were the same. Risk ratio was used as the measure for treatment effect. Outcomes of interest related to efficacy and safety.

**Findings:**

Of the 5441 total records identified by our searches, nineteen studies (6332 patients, 31.0% female) were eligible for the systematic review and twelve (3763 patients, 31.0% female) for meta-analysis. Rifampicin doses varied from 8 to 35 mg/kg and implementation of the intervention varied between trials. There was no evidence for increased efficacy with higher doses of rifampicin, however the majority of trials investigated minimally increased doses (up to 20 mg/kg). At higher doses (>20 mg/kg), there may be evidence of increased risk of drug-induced liver injury, albeit with no consistent dose–response relationship.

**Interpretation:**

Evidence on the efficacy of higher doses of rifampicin in the first-line regimen for TB remains incomplete. While higher doses appear generally safe, the risk of drug-induced liver injury may be increased above doses of 20 mg/kg. Larger clinical trials reporting definitive outcomes are needed to determine whether dosing up to 40 mg/kg could safely improve treatment outcomes or reduce duration of first-line therapy.

**Funding:**

10.13039/100004423WHO, 10.13039/100010269Wellcome Trust.

**Research in context**

**Evidence before this study**

Despite rifampicin being the most important drug in the first-line regimen for drug-susceptible TB, the pharmacological basis of this fundamental role is not fully understood. Rifampicin demonstrates high inter-person and inter-study variability in pharmacokinetic studies, however there is evidence that higher doses result in higher exposure in plasma and/or cerebrospinal fluid. Studies also suggest that doses up to 40 mg/kg may be tolerable in humans and the current recommended dose (8–12 mg/kg) may not maximise the potential benefits of this drug. Two previous systematic reviews investigated increased doses of rifampicin, both focusing exclusively on pulmonary TB and reporting the main outcome as the intermediate endpoint of culture conversion. One of these reviews described some longer-term outcomes, finding no difference in mortality or moderate to severe liver toxicity between standard and higher dose groups. This is an updated version of a WHO commissioned review published in the WHO operational handbook on tuberculosis (2022 update).

**Added value of this study**

To our knowledge, this is the first systematic review and meta-analysis reporting on the efficacy and safety of higher dose rifampicin in adults with any type of presumed drug-susceptible TB. The review focused on key long-term outcomes and study settings were diverse in terms of both geographical location and TB incidence. Many countries with the highest TB rates were represented, with the notable exception of China. Despite diverse designs and reported outcomes in the eighteen included trials, meaningful data synthesis was possible for key objectives. Higher than currently recommended doses of rifampicin appear to be safe and may be associated with reduced rates of treatment failure, relapse and all-cause mortality in pulmonary and meningeal TB.

**Implications of all the available evidence**

Larger clinical trials reporting definitive outcomes are needed to determine whether rifampicin doses up to 40 mg/kg could safely improve treatment outcomes or reduce duration of first-line therapy. The limited data does not permit identification of any important subgroups (e.g., those with HIV-associated TB or diabetes) that might benefit from this intervention. Drug–drug interaction studies are essential to understand the robustness of antiretroviral therapy and facilitate enrolment of more people with HIV-associated TB into future trials. Ongoing and planned Phase III randomised controlled trials of high-dose rifampicin in pulmonary, meningeal and disseminated TB will increase certainty of evidence at the higher doses examined in this review.

## Introduction

Tuberculosis (TB) remains a pressing public health issue, with 1.3 million deaths worldwide and incident cases increasing to 10.6 million in 2022.^1^ While 85% of people successfully complete first-line treatment comprising rifampicin (RIF), isoniazid, pyrazinamide and ethambutol, this regimen requires six months to achieve durable cure, representing a significant burden for people with TB and their carers.

Of first-line treatment, RIF is the cornerstone. Mono-resistance to RIF is independently associated with the highest risk of treatment failure (RR 5.5)^2^ and duration of RIF administration is a key determinant of risk of relapse.^3^ This fundamental role is not fully understood but may be due to activity against non-replicating, antibiotic-tolerant *Mycobacterium tuberculosis* organisms^4^^,^^5^ and accumulation in pulmonary TB lesions.^6^

Mycobacterial killing is believed to be driven by the parameter area under the curve of RIF plasma concentration divided by Minimum Inhibitory Concentration of infecting organism (AUC/MIC). However, plasma concentrations of RIF exhibit high inter-person and inter-study variability.^7^ AUC/MIC values achieved on current doses of RIF vary more than three-fold^8^ and are on average much lower than those predicted to be optimal in preclinical systems, especially in the cerebrospinal space.^9^ This evidence suggests increased dosing of RIF could plausibly increase rates of treatment success. Whether these potential benefits can be realised in long-term outcomes and be achieved without additional toxicity has not been established.

RIF has traditionally been administered at a dose of 10 (range 8–12) mg/kg, based on pragmatic cost- and risk-benefit considerations. Perceptions of immune-mediated side effects and of drug-induced liver injury (DILI) may have limited exploration of higher dose levels.^10^ More recently, renewed study of dose and concentration-response relationships has caused re-evaluation of dosing schedules. In pre-clinical models, humanised levels of pharmacokinetic (PK) exposure do not maximise elimination of bacilli.^11^ Early phase trials have shown evidence of incremental dose–response beyond the current dose range,^12^ confirmed in two recent Phase IIB trials of pulmonary disease.^8^^,^^13^ Modestly higher doses of RIF have also been evaluated in extrapulmonary disease, particularly meningeal TB.^14^^,^^15^ Two phase III trials of higher dose RIF in pulmonary TB have recently been reported.^16^^,^^17^ Studies have not to date reported increased risk of serious adverse events (SAEs) such as hepatotoxicity at higher doses. Souleymane et al.^18^ recently stopped recruitment to the intervention arm (30 mg/kg RIF and high-dose isoniazid) of the TRIDORE RCT owing to significantly higher rates of adverse drug reactions in this arm. Given the addition of high-dose isoniazid, these AEs cannot be solely attributed to RIF. Phase II and III trials aiming to optimise higher dosing of RIF in pulmonary TB, HIV-associated TB and in tuberculous meningitis are ongoing (Appendix 1).

Maximising the efficacy of the first-line regimen is key to improving long-term outcomes of TB treatment and ensuring robustness against variability in adherence, PK, pharmacogenetics and resistance emergence. Intensification of treatment could also be important for those with severe or disseminated disease and higher RIF doses could ultimately reduce the duration of first-line treatment, which is amongst the objectives of a number of included studies.

Our primary objective is to assess the efficacy and safety of doses of RIF higher than those currently recommended by the World Health Organisation (WHO) when used as part of a combination regimen for treating adults with presumed drug-susceptible TB.

## Methods

This systematic review and meta-analysis was reported in line with the PRISMA statement.^19^ This review was not registered.

### Search strategy and selection criteria

We used the same search strategy as in our previous review,^20^ searching for eligible trials in MEDLINE (OVID), EMBASE (OVID), CENTRAL (Cochrane central register of controlled trials), WHO International Clinical Trials Registry and Clinicaltrials.gov, with no limits for language, date of publication or publication status (Appendix 2). Searches were run up to 31 July 2024 by a Cochrane information specialist (VL). Reference lists of retrieved reports were examined for unidentified studies. Active investigators in the field were contacted to provide information on any unidentified, ongoing or planned trials.

Inclusion criteria were randomised controlled trials of adults with presumed drug-sensitive TB (pulmonary, extrapulmonary, disseminated) on first-line treatment. Adults were defined as aged 18 years or over, or treated as adults in participating centres. Where it was not possible to differentiate data of participants under 18 years, the younger minority were included.

Assessed interventions were TB treatment regimens of any duration containing RIF at doses higher than recommended in current WHO guidelines (8–12 mg/kg). The comparator was treatment regimens containing RIF at recommended doses. Inclusion for meta-analysis required the same background regimen alongside RIF. Randomised concentration-controlled trials were not within the scope of this review. Although not recommended by WHO guidance, intravenous administration of RIF was permissible. The dose metric used was actual weight-adjusted dose in mg/kg. Where not specified, this was determined using weight data provided. Where weight-banded dosing regimens were used, average target weight-adjusted dose stated in the report of manufacturer’s summary of product characteristics was accepted or, if not specified, the average of weight-adjusted doses computed using the midpoint of each band.

Search results were uploaded to the Covidence interface^21^ and de-duplicated. Two reviewers independently screened titles and abstracts against inclusion and exclusion criteria. Two authors (KAH, HHT) independently screened full-text study reports. Reasons for non-inclusion were documented, with disagreements resolved by discussion, or the assistance of a third author (GD).

### Data analysis

A data extraction form was designed, piloted and optimised. Two authors (KAH, HHT) independently extracted data. Where appropriate, multiple reports on the same study were collated. Data extraction completion was verified by two authors (KAH, HHT, LB, PEN). Data extraction included details on source, methods, participants, microbiological methods, pharmacology, interventions and outcomes (Appendix 3).

Primary outcomes were treatment success, treatment failure, relapse, death and adverse events. Secondary outcomes were SAEs, drug-specific adverse events of interest and disease-specific efficacy outcomes of interest. Definitions of outcomes can be found in appendices (Appendix 4). Summary data were extracted, where available, to enable intention to treat (ITT) and per protocol (PP) analyses.

Extracted data was imported into R version 4.3.3^22^ for analysis. Meta-analysis was performed with random effects models.^23^ Risk ratio (RR) was used as the measure of effect. Analysis of the primary outcomes was on an ITT basis. For studies where more than one intervention arm was included, each arm was compared separately to the comparator to avoid splitting the control group. Stratified forest plots were used to present data. Corresponding 95% confidence intervals and p-values were computed with a significance level of 0.05. Heterogeneity was assessed by inspecting forest plots and, for meta-analysis, using the Iˆ2 statistic, with a value of 50% taken to indicate significant statistical heterogeneity.

Two authors independently assessed the methodological quality of each study using the Cochrane risk of bias tool.^24^ A threshold of 90% was set for adequate follow-up of randomised participants. We attempted to contact study authors if information was unspecified or unclear. Funnel and Galbraith plots were inspected for evidence of publication bias. Summary of findings tables were constructed to present certainty of evidence ratings for effect estimates for each outcome along with relative and absolute measures of effect using the GRADE approach.^25^

### Role of the funding source

The funding source had no role in study design, data collection, analysis, interpretation or writing of the report.

## Results

**Fig. 1:**
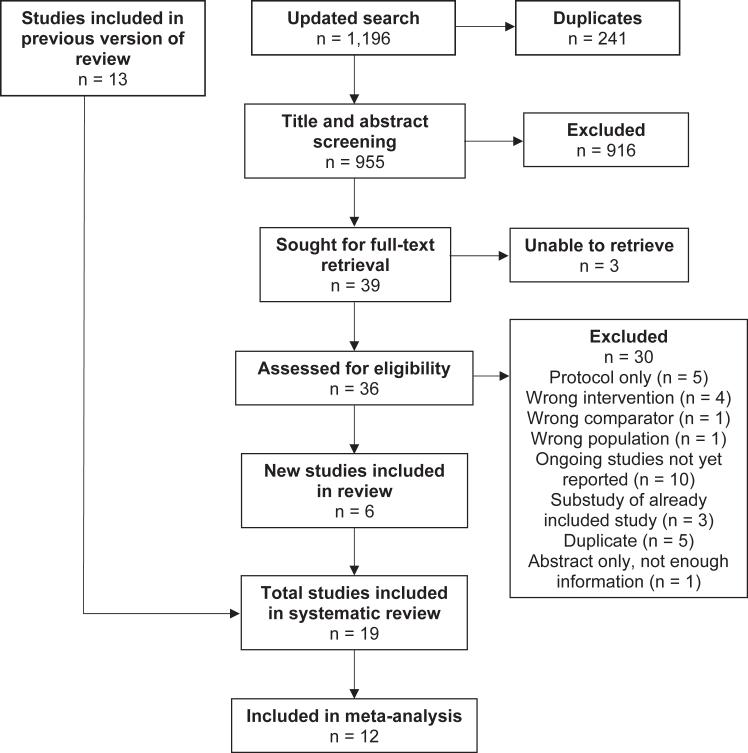
PRISMA diagram. Table of excluded studies in Appendix 5.

Searches identified 1196 records, reduced to 955 after deduplication, and to 39 after title and abstract screening. After full-text review, a further six studies were included (Fig. 1).

Eligible studies were published between 1979 and 2024 and included 6332 participants recruited from inpatient and outpatient settings in Africa,^13^^,^^16^^,^^17^^,^^26^, ^27^, ^28^, ^29^, ^30^, ^31^, ^32^ Asia,^14^, ^15^, ^16^, ^17^^,^^31^^,^^33^, ^34^, ^35^, ^36^, ^37^ South America^8^^,^^17^^,^^31^ and North America^38^(Table 1). All except one^32^ were in full paper format. Most (n = 14) included participants of 18 years or above, but some included participants of 14 years or above,^38^ 15 years or above^14^^,^^34^^,^^35^ and 17 years or above.^33^

**Table 1:** Characteristics of included studies.

	Setting	Comparator RIF dose (mg/kg)	Intervention RIF dose (mg/kg); other intervention	Participants, total (n = female)	Controls	Type of TB	TB diagnosis	Length of RIF intervention	HIV status (positive, n (%))	Included in meta-analysis
Aarnoutse et al. (2017)^29^	Inpatients, outpatients; Tanzania	10	15, 20	150 (15)	50	Pulmonary	ZN stain (confirmed with AccuProbe, LJ or MGIT)	2/12	15 (10%), unclear if all tested	Yes
Atwine et al. (2020)^30^	Unclear; Uganda	10	20; different doses of efavirenz	98 (27)	33	Pulmonary	GeneXpert MTB/RIF	2/52	98 (100%), inclusion criterion	Yes
Boeree et al. (2017)^13^	Inpatients, outpatients; Tanzania, South Africa	10	35, 20 + SQ109, 20+moxifloxacin, 10 + SQ109	365 (107)	123	Pulmonary	ZN stain, GeneXpert MTB/RIF	12/52	24 (6.6%), all tested	Yes; arms with comparable background regimens only
Cresswell et al. (2021)^27^	Inpatients; Uganda	10	35, 20 (IV) then 35	61 (27)	21	Meningeal	Clinical diagnosis; CSF glucose to plasma <50% or CSF glucose <65 mg/dl or positive CSF AFB smear or positive GeneXpert MTB/RIF or Ultra	8/52	56 (91.8), all tested	Yes
Davis et al. (2023)^26^	Inpatients; South Africa	10	35+linezolid, 35+linezolid + aspirin	52 (22)	20	Meningeal	Definite, probable or possible TBM	56/7	52 (100%),	No; different background regimens between control and intervention arms
Dian et al. (2018)^34^	Inpatients; Indonesia	10	20, 30	60 (28)	20	Meningeal	Clinical diagnosis; CSF/blood glucose ratio <0.5	1/12	6 (10%), all tested	Yes
Heemskerk et al. (2016)^15^	Inpatients; Vietnam	10	15+levofloxacin	817 (257)	409	Meningeal	Clinical diagnosis; 5/7 symptoms, nuchal rigidity, CSF abnormal	2/12	349 (42.7%), all tested	No; different background regimen between control and intervention arms
Jindani et al. (2016)^31^	?outpatients; Bolivia, Nepal, Uganda	10	15, 20	300 (95)	100	Pulmonary	2x sputum ZN stain	16/52	0 (0%), all tested	Yes
Jindani et al. (2023)^17^	Outpatients; Uganda, Guinea, Peru, Nepal, Botswana, Pakistan	10	∼23 (1200 mg), ∼35 (1800 mg)	672 (154)	224	Pulmonary	GeneXpert MTB/RIF	4/12	0 (0%), exclusion criterion	Yes
Kannabiran et al. (2024)^37^	Unclear; India	10	25, 35	333 (96)	109	Pulmonary	GeneXpert MTB/RIF, LJ, MGIT	8/52	0 (0%), exclusion criterion	Yes
Long et al. (1979)^38^	Inpatients, outpatients; USA	∼8 (450 mg)	∼11 (600 mg), ∼13 (750 mg)	822 (176)	167	Pulmonary	AFB on sputum microscopy; CXR suggestive	20/52	Not documented	No; not comparable with any other study and provided data inconsistent
Maug et al. (2020)^35^	Outpatients; Bangladesh	10	20	701 (187)	348	Pulmonary	Smear positive	6/12	Not tested, known HIV excluded	Yes
Merle et al. (2016)^32^	Unclear; Benin, Guinea, Senegal	10	15; ART initiation at 2/52 or 8/52	778 (339)	262	Unclear; sputum samples taken, assume pulmonary	Bacteriologically confirmed TB	2/12	778 (100%), inclusion criterion	Yes
Paton et al. (2023)^16^	Outpatients; Indonesia, Philippines, Thailand, Uganda, India	10	35 (reduced to 20 during trial) + linezolid, 35 (reduced to 20 during trial) + clofazimine; rifapentine + linezolid + levofloxacin; bedaquilline + linezolid	675 (254)	181	Pulmonary	GeneXpert MTB/RIF; symptoms of TB or CXR suggestive	2/12	0 (0%), originally exclusion criterion	No; different background regimens between control and intervention arms
Ruslami et al. (2007)^36^	Outpatients; Indonesia	10	13	50 (24)	25	Pulmonary	Microscopy; CXR suggestive	6/12	At least 1 (0.5%), but unclear; all tested	No; not comparable with any other study
Ruslami et al. (2013)^14^	Inpatients; Indonesia	10	13 (IV); moxifloxacin two doses; ethambutol	60 (27)	12	Meningeal	Definite, probable or possible TBM	14/7	7 (11.7%), all tested	No; different background regimen between control and intervention arms
Sekaggya-Wiltshire et al. (2022)^28^	Outpatients; Uganda	10+dolutegravir, 10+efavirenz	35+dolutegravir, 35+efavirenz,	128 (47)	67	Pulmonary	GeneXpert MTB/RIF, urine LAM, sputum culture	8/52	(128), all tested	Yes
Velasquez et al. (2018)^8^	Outpatients; Peru	10	15, 20	180 (66)	60	Pulmonary	Smear positive	8/52	5 (2.8%), all tested	Yes
Yunivita et al. (2016)^33^	Inpatients; Indonesia	13 (IV)	17, 20	30 (12)	10	Meningeal	Definite, probable or possible TBM	14/7	6 (20%), all tested	No; not comparable with any other study

Studies predominantly recruited participants with pulmonary (n = 13) or meningeal (n = 6) TB. HIV infection was documented in all but one^38^ study. Three studies recruited only people living with HIV.^26^^,^^30^^,^^32^ One study did not test all participants, but excluded known HIV positive individuals.^35^ Across other trials the range of HIV coinfection varied from 0 to 99%. Where HIV was reported, 27.7% (1525/5510) of participants were living with HIV.

All but two reports presented doses of RIF in mg/kg format. For Long 1979^38^ and Jindani 2023,^17^ mg/kg values were estimated using weight band data provided. Evaluated doses varied from 8 mg/kg to 35 mg/kg. Seventeen trials compared the standard 10 mg/kg dose to higher doses. One used 13 mg/kg given intravenously (IV)^33^ as the lower dose. Another compared oral 10 mg/kg to IV 13 mg/kg.^14^ Data from oral versus IV doses were not included in meta-analysis but reported separately where appropriate. Treatment was directly observed for the whole intervention period in twelve studies. Length of follow-up varied considerably from two to ninety-six weeks. Length of intervention varied from two weeks to six months. Data from the Jindani 2023^17^ estimated 23 mg/kg arm and the Kannabiran 2024^37^ 25 mg/kg arm were pooled for meta-analysis as variability of dosing within weight bands between both studies approximately equates to 22–27 mg/kg.

Ten studies provided treatment success data.^8^^,^^13^^,^^17^^,^^28^, ^29^, ^30^, ^31^, ^32^^,^^35^^,^^38^ Numerous trial-specific definitions of treatment success were used by investigators (Appendix 6). There was a high certainty of no evidence for higher rates of treatment success at RIF doses of 15 mg/kg (RR 1.00 [0.97–1.03], RD −1.0% [−5.5% to 3.5%], 4 trials, 916 participants) and 20 mg/kg (RR 1.01 [0.97–1.06], RD 2.9% [−1.1% to 7.0%], 6 trials, 1302 participants) and a modest reduction at 23 mg/kg (RR 0.96 [0.91–1.03], RD −3.2% [−8.9% to 2.4%], 1 trial, 373 participants) and 35 mg/kg (RR 0.97 [0.90–1.04], RD −6.1% [−13.8% to −1.6%], 2 trials, 316 participants) (Appendix 7; analyses 1–3). Jindani 2023 evaluated 35 mg/kg at a shorter duration of four months and did not conclude non-inferiority.^17^ Similar results were obtained for per protocol analyses (Appendix 6; analysis 4). Long 1979^38^ used distinct dose levels from other included studies, thus data was not combined; the trial found no statistically significant difference between estimated 10 mg/kg and 13 mg/kg doses.

Nine studies provided treatment failure data^8^^,^^17^^,^^28^, ^29^, ^30^^,^^32^^,^^35^^,^^36^^,^^38^ although there was a lack of consistency of definitions used by investigators (Appendix 6). Four studies^17^^,^^28^^,^^35^^,^^36^ defined treatment failure in line with our definition, based on sputum smear positivity from month five onwards. Two studies^8^^,^^32^ did not specify a definition of treatment failure but, as these are recent studies of patients with pulmonary TB, are assumed to have used the WHO definition. The evidence suggests RIF doses of 15 mg/kg may reduce treatment failure (RR 0.71 [0.27–1.88], RD −1.0% [−3.6% to 1.6%], 2 trials, 616 participants), doses of 20 mg/kg may result in no difference (RR 1.01 [0.29–3.61], RD 0.5% [−1.9% to 2.8%], 2 trials, 821 participants) and doses of 23 mg/kg may increase treatment failure (RR 4.52 [0.99–20.66], RD 3.8% [0.3%–7.2%], 1 trial, 375 participants) (Appendix 7; analysis 5).

Five trials reported relapse rates.^8^^,^^17^^,^^32^^,^^35^^,^^38^ The minimum follow-up time for these studies was one year. RIF doses of 15 mg/kg (RR 0.79 [0.25–2.45], RD −0.7% [−2.8% to 1.5%], 2 trials, 617 participants) and 20 mg/kg (RR 0.93 [0.07–12.72], RD −0.0% [−1.2% to 1.2%], 2 trials, 821 participants) may reduce risk of relapse (Appendix 7; analysis 6). Jindani 2023^17^ provided relapse data for mITT population with very low numbers of events leading to wide confidence intervals (Appendix 7; analysis 7).

**Fig. 2:**
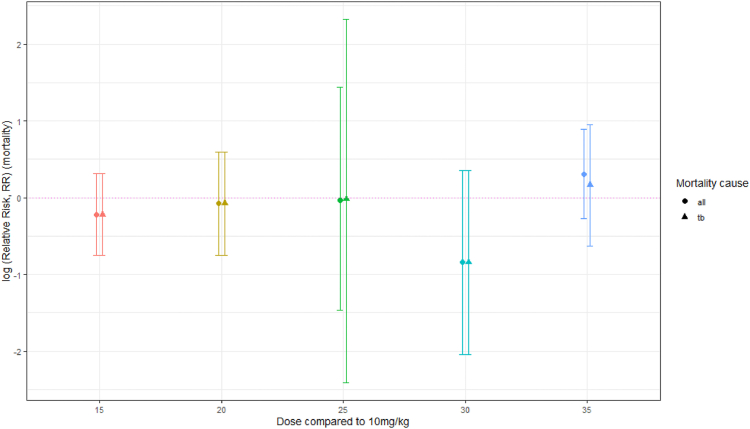
Summary plot of mortality RR per comparison group for all-cause versus TB mortality (log (RR and 95% confidence intervals), dotted horizontal line RR = log (1)).

All but one study^33^ provided mortality data. The evidence suggests RIF doses of 15 mg/kg (RR 0.80 [0.47–1.37], RD −1.1% [−4.0% to 1.8%], 4 trials, 916 participants) and 20 mg/kg (RR 0.93 [0.47–1.81], RD −0.4% [−2.2% to 1.3%], 7 trials, 1342 participants) probably reduce all-cause mortality. Similar results were obtained for PP analysis. Eight studies compared doses higher than 20 mg/kg with control; six had the same background regimens allowing for data synthesis. Evidence across these studies is uncertain owing to small numbers of participants or events (<5%; 32 events, 837 participants). Doses of 25 mg/kg (RR 0.97 [0.23–4.22], RD 0.3% [−2.1% to 2.6%], 2 trials, 780 participants) and 30 mg/kg (RR 0.43 [0.13–1.43], RD −20.0% [−46.1% to 6.1%], 1 trial, 40 participants) may reduce all-cause mortality compared to control. Doses of 35 mg/kg may increase all-cause mortality although the evidence is uncertain (RR 1.35 [0.76–2.43], RD 1.4% [−0.8% to 3.7%], 5 trials, 1139 participants) (Appendix 7; analyses 8–11). No clear dose–response relationship is evident (Fig. 2).

Velasquez 2018^8^ was the only study with no participant mortality. Sekaggya-Wiltshire 2023,^28^ Jindani 2023^17^ and Kannabiran 2024^37^ specified causes of death that were not TB-related. Assuming all other deaths were TB-related, analysis at 25 mg/kg (RR 0.98 [0.09–10.17], RD 0.6% [−1.4% to 2.6%], 2 trials, 780 participants) suggests TB-related mortality may reduce, albeit with wide confidence intervals owing to low numbers of events, but at 35 mg/kg (RR 1.18 [0.53–2.60], RD 0.1% [−1.7% to 1.9%], 5 trials, 1139 participants) there may be increased TB-related mortality (Fig. 2; Appendix 7, analysis 12).

Adverse event reporting was consistent across all studies, albeit with great variability in data presentation. Several studies reported only hepatotoxicity-related adverse events. No studies reported statistically significant difference in adverse events between groups. Studies were not comparable by meta-analysis owing to background regimen variability.

**Fig. 3:**
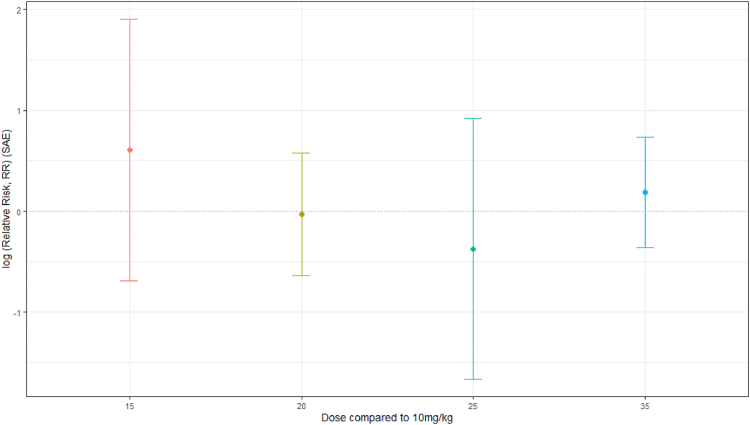
Summary plot of SAE RR per comparison group (log (RR and 95% confidence intervals), dotted horizontal line RR = log (1)).

Ten studies provided SAE data.^8^^,^^13^^,^^16^^,^^17^^,^^27^, ^28^, ^29^, ^30^, ^31^^,^^35^^,^^37^ For RIF doses of 20 mg/kg (RR 0.97 [0.53–1.78], RD −0.9% [−3.3% to 1.5%], 6 trials, 1302 participants) and 25 mg/kg (RR 0.69 [0.19–2.51], RD −0.6% [−2.5% to 1.2%], 2 trials, 780 participants) the evidence suggests there may be a decrease in SAEs, while for doses of 15 mg/kg (RR 1.83 [0.50–6.69], RD 1.4% [−1.3% to 4.2%], 3 trials, 417 participants) and 35 mg/kg (RR 1.21 [0.70–2.09], RD 0.8% [−1.8% to 3.3%], 5 trials, 1139 participants) higher doses may increase SAEs, but the evidence is very uncertain and there is no clear dose response (Fig. 3; Appendix 7, analyses 13–14).

**Fig. 4:**
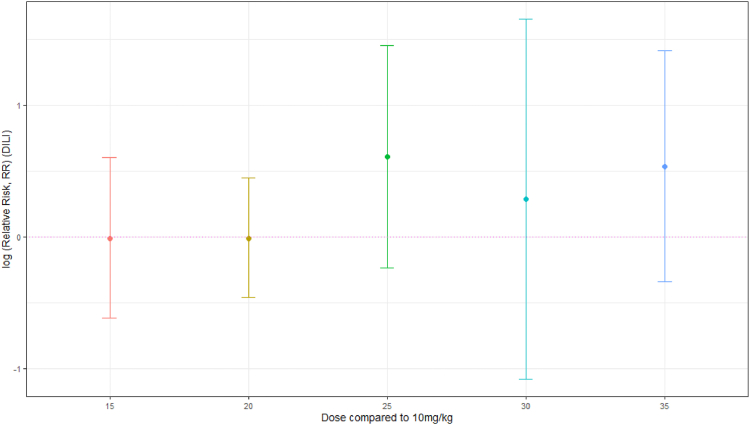
Summary plot of drug-induced liver injury RR per comparison group (log (RR and 95% confidence intervals), dotted horizontal line RR = log (1)).

All but one study^38^ reported DILI. However, the majority of authors did not specify whether they felt DILI was due to RIF or other potentially hepatotoxic concomitant medications. The evidence suggests that there is little or no difference in risk of DILI at RIF doses of 15 mg/kg (RR 0.99 [0.54–1.83], RD 1.1% [−2.9% to 5.0%], 4 trials, 589 participants) or 20 mg/kg (RR 0.99 [0.63–1.56], RD 0.0% [−2.3% to 2.3%], 7 trials, 1342 participants). Data synthesis suggests, with uncertainty, that doses of 25 mg/kg (RR 1.84 [0.79–4.27], RD 2.0% [−0.7% to 4.8%], 2 trials, 780 participants), 30 mg/kg (RR 1.33 [0.34–5.21], RD 5.0% [−18.5% to 28.5%], 1 trial, 40 participants) and 35 mg/kg (RR 1.70 [0.71–4.10], RD 3.3% [0.7%–5.9%], 5 trials, 1139 participants) may increase risk of DILI, although no clear dose–response relationship is apparent (Fig. 4; Appendix 7, analyses 15–17).

Five studies reported treatment success and/or mortality data but were not appropriate to be included in meta-analysis or pooled estimation owing to variance in background regimens, with the exception of Boeree 2017,^13^ where some intervention arms were included in meta-analysis where background regimens were comparable. RR of reported outcomes in these studies are in keeping with cumulative estimates from studies included in meta-analyses, including for studies where fluoroquinolones were an additional intervention in the context of higher RIF doses (Appendix 8).

Reporting of other secondary outcomes can be found in the appendices (Appendix 9).

Risk of bias for efficacy and safety outcomes were separated and tables generated (Appendix 10).^39^ Risk of bias for both efficacy and safety results was generally unclear to high, predominantly owing to the open-label nature of many included trials. Detail on bias interpretation can be found in Appendix 10.

## Discussion

This review addresses whether higher doses of RIF than currently recommended could safely improve outcomes of first-line TB treatment in adults. Despite diverse designs and reported outcomes in the 19 included trials, meaningful data synthesis was possible. Doses studied ranged from 8 to 35 mg/kg with some variation of companion regimens. Most included trials were Phase IIB or small Phase III superiority trials with no difference in duration of arms. RIFASHORT^17^ was the only included non-inferiority trial, a relatively small trial that had a shorter duration of intervention, which may have potentially biased against the intervention in a pooled analysis. With the notable exception of Long 1979,^38^ trials were published within the last sixteen years. Studies reporting the highest doses of 30 mg/kg^34^ and 35 mg/kg^13^^,^^16^^,^^17^^,^^26^, ^27^, ^28^^,^^37^ were published within the last seven years.

RIF doses above 10 mg/kg were not associated with higher rates of treatment success. Higher doses did not appear to be associated with any change in rate of treatment failure or relapse, but reported numbers of events were low. Efficacy results >20 mg/kg were dominated by RIFASHORT,^17^ as such there is minimal evidence on whether treatment success would improve at a duration of six months, but there is a low certainty of evidence that the intervention at four months has worse outcomes. There is some evidence that doses of RIF higher than 10 mg/kg may reduce all-cause mortality, but there is substantial uncertainty and dose–response relationships were inconsistent. Furthermore, most data derive from trials using doses up to 20 mg/kg. Trials with shorter follow-up periods may have missed later mortality.

During first-line TB therapy, hepatotoxicity may occur at an incidence of 1% or more and has been a rationale for caution against RIF dose escalation.^10^ In this review, inconsistent safety reporting limited data synthesis and interpretation of adverse events was complicated by diversity of background regimens. There was no clear evidence to suggest higher rates of SAEs with higher doses of RIF (Fig. 3). However, the data suggest, with uncertainty, that doses of RIF higher than 20 mg/kg may be associated with increased risk of DILI, albeit with no evident dose–response relationship between 25 and 35 mg/kg (Fig. 4). It has been suggested that RIF-induced DILI could be an idiosyncratic reaction.^40^

Study settings were diverse. Many countries with the highest TB rates were represented, with the notable exception of China. Trials focussed on pulmonary and meningeal TB. Disseminated or other forms of extrapulmonary TB were not represented. People living with HIV were relatively under-represented. Trial investigators frequently imposed restrictions on use of antiretroviral therapy to avoid drug–drug interactions. Most trials were conducted in settings where HIV was uncommon, excluded people with HIV, or allowed enrolment only if baseline CD4 count was >200 cells/mmˆ3. Diabetes did not form part of the rationale of any study and was rarely reported on, despite being associated with poorer outcomes.^41^ These restrictions limit the immediate applicability of the evidence in settings with high HIV incidence or for people with diabetes.

The review focused on programmatically important outcomes. As many trials were smaller Phase II studies, the outcomes of interest were frequently not reported or below the Optimal Information Size. This was reflected in broad confidence intervals, typically including substantial benefit and harm. However, heterogeneity as measured by the Iˆ2 statistic was uniformly low across almost all analyses. Direct comparison was not possible between a number of studies owing to varying background regimens, as investigators frequently aimed to trial a novel regimen with potentially increased potency rather than focusing on defining RIF dose–response (Appendix 8). There is not currently adequate evidence on higher doses of RIF with key companion drugs such as fluoroquinolones. Further evidence on these interventions with a range of durations would be desirable.

Certainty of evidence has been summarised using the GRADE approach in a Summary of Findings table (Appendix 11). Problems with risk of bias were identified, particularly regarding safety outcomes (Appendix 10). This mainly resulted from open-label methodology; although authors did not judge blinding to be feasible, predominantly due to the likelihood of increased discolouration of bodily fluids with higher doses of RIF, the risk of performance and detection bias remains. To limit bias in the review process, measures outlined in the Cochrane group were followed.^24^ No publication bias was detected for key outcomes (Appendix 12).

Several early phase studies claimed evidence of improved efficacy at higher doses on the basis of investigator-defined endpoints, typically measures of culture conversion at eight or twelve weeks of therapy. Compared to 10 mg/kg, Boeree 2017^13^ concluded that a RIF dose of 35 mg/kg reduced time to culture conversion, Jindani 2016^31^ reported an increase in culture conversion at 20 mg/kg (p = 0.09), Kannabiran 2024^37^ reported increased culture conversion at 25 mg/kg (p < 0.001), Sekaggya-Wiltshire 2022^28^ and Kannabiran 2024^37^ both reported increases in culture conversion at 35 mg/kg (p = 0.063 and p < 0.001 respectively) and Velasquez 2018^8^ demonstrated dose–response at 15 and 20 mg/kg based on statistical modelling of sputum colony counts. However, whether improvements in culture status at early timepoints translate consistently into better long-term outcomes is unclear.

The scope of this review was restricted to regimens as similar as possible to the current recommended first-line regimen, as such studies of intermittent high-dose RIF regimens were excluded. Such regimens have typically been designed to deliver a cumulative weekly dose similar to daily regimens and, as PK-PD interactions between dose size and interval for RIF are not completely characterised, these studies may have been difficult to interpret.

Some included trials were either primarily PK studies or had nested PK studies. All but one of these^33^ reported higher doses of RIF consistently resulted in higher exposure in plasma and/or cerebrospinal fluid.^29^^,^^34^^,^^36^^,^^37^ PK studies not included in this review report that higher PK exposures may be tolerable in humans at doses up to 40 mg/kg, suggesting scope for future trials at higher doses.^40^ We note that studies of newer rifamycins routinely include comparatively higher doses than are typically used in rifampicin studies (e.g., study 31^42^).

Two previously published systematic reviews investigating high dose RIF were identified. Both focused solely on pulmonary TB.^43^^,^^44^ Steingart 2011 reported higher doses of RIF resulted in improved culture conversion rates, advocating for clinical trials to confirm efficacy and tolerability.^44^ Onorato 2021 reported that higher doses of RIF were associated with increased rate of sputum culture conversion, particularly at doses of 20 mg/kg or above. No difference was detected in mortality between treatment groups and similar rates of hepatotoxicity were observed.^43^ Although these reviews focused more on intermediate bacterial endpoints than longer-term outcomes such as treatment success, findings agree with the conclusions of our review, particularly with regard to safety outcomes.

Four systematic reviews investigating RIF PK were identified. Two reported that patients often had subtherapeutic drug concentrations of RIF.^7^^,^^45^ Two more recently reported that low concentrations of RIF appear to be related to poor treatment outcome.^46^^,^^47^ These reviews strengthen the rationale for higher RIF doses in individualised therapy and suggest the possibility that globally higher doses might reduce the risk that an important subgroup could fall below target PK-PD thresholds.

The main limitations of this systematic review and meta-analysis are the small numbers of studies in some dosing brackets, the variability across definitions of treatment success and the risk of bias. In addition, funnel plots should ideally be utilised when there are at least ten studies. In the absence of a better tool, we used funnel plots to provide an estimation of publication bias.

In conclusion, we did not find evidence that RIF doses higher than the recommended 10 mg/kg were associated with higher rates of treatment success in pulmonary and meningeal TB, although they may be associated with reduced rates of treatment failure, relapse and all-cause mortality. No important differences were found in safety outcomes and tolerability, with the exception of DILI, where doses >20 mg/kg may be associated with increased risk. The evidence for these outcomes is uncertain and there was no consistent pattern of dose–response. This review does not therefore provide direct support for increasing RIF dosing for all patients in routine practice, but suggests that increased dosing appears safe. The limited data does not permit identification of any important subgroups that might benefit from the intervention. Larger clinical trials reporting definitive outcomes are needed to determine whether RIF doses up to 40 mg/kg could safely improve treatment outcomes or reduce duration of first-line therapy. Results of ongoing and planned Phase III RCTs of high-dose RIF in pulmonary, meningeal and disseminated TB will increase certainty of evidence at the higher doses examined in this review.

## Contributors

GD and KAH were responsible for conceptualisation of this work. GD, KAH, VL and SN developed methodology. KAH and HHT screened articles. KAH and HHT extracted data; KAH, HHT, LB and PEN reviewed extracted results. Statistical analysis was undertaken by KAH with support from SN. Original manuscript was prepared by KAH with support from GD. All authors had full access to data and reviewed final manuscript, sharing responsibility for the decision to submit for publication.

## Data sharing statement

Data collected for this study and R code will be made available to others on request by email to the corresponding author.

## Declaration of interests

GD was supported by a consultancy contract from WHO for the initial published version of this review and chaired the Data Safety Monitoring Board for RIFASHORT and the Trial Steering Committee for TRUNCATE-TB. All other authors declare no competing interests.

## Appendix ASupplementary data

Appendices 1–12
