# Supplementary material for: Efficacy and safety of higher dose rifampicin in adults with presumed drug-susceptible tuberculosis: an updated systematic review and meta-analysis
Source: eClinicalMedicine. 2024 Oct 3;77:102857. doi: 10.1016/j.eclinm.2024.102857 (PMC11474450; doi:10.1016/j.eclinm.2024.102857)

**Appendix 1**

Ongoing RCTs using high dose RIF in adults with presumed drug-susceptible TB

| **Trial** | **Phase** | **Type of TB** | **Intervention dose of RIF** | **Identifier** |  |
| --- | --- | --- | --- | --- | --- |
| New-Strat TB | III | HIV-associated disseminated | 35mg/kg | NCT04951986 |  |
| DATURA | III | HIV-associated (any TB) | 35+/-5mg/kg | NCT04738812 |  |
| INTENSE-TBM | III | TBM | 35mg/kg | NCT04145258 |  |
| HARVEST | III | TBM | Additional 1200mg to normal weight banded dosing | ISRCTN15668391 |  |
| STEP2C | IIB/C | Pulmonary | Fixed 2100mg | NCT05807399 |  |
| A5384 | II | TBM | 35mg/kg | NCT05383742 |  |
| IMPI-3 | II | Tuberculous pericarditis | 35mg/kg | NCT04521803 |  |
| OptiRiMoxTB | III | Pulmonary | 35mg/kg | NCT05575518 |  |

**Appendix 2: Search strategy**

Ovid MEDLINE(R) ALL <1946 to March 22, 2023>

1 (tuberculosis or tuberculous ot TB or MDR-TB or XDR-TB).tw.

2 Tuberculosis/

3 1 or 2

4 rifampin.mp. or Rifampin/

5 rifampicin.tw.

6 ethambutol.mp. or Ethambutol/

7 isoniazid.mp. or Isoniazid/

8 pyrazinamide.mp. or Pyrazinamide/

9 4 or 5 or 6 or 7 or 8

10 3 and 9

11 randomized controlled trial.mp.

12 controlled clinical trial.mp.

13 (randomized or randomly or placebo or groups).tw.

14 11 or 12 or 13

15 10 and 14

16 exp Animals/

17 Humans/

18 16 not 17

19 15 not 18

20 10 and 19

Embase 1947-Present, updated daily

1 (tuberculosis or tuberculous ot TB or MDR-TB or XDR-TB).tw.

2 Tuberculosis/

3 1 or 2

4 rifampin.mp. or Rifampin/

5 rifampicin.tw.

6 ethambutol.mp. or Ethambutol/

7 isoniazid.mp. or Isoniazid/

8 pyrazinamide.mp. or Pyrazinamide/

9 4 or 5 or 6 or 7 or 8

10 3 and 9

11 (random* or factorial* or placebo* or assign* or allocat* or crossover*).tw.

12 ((blind* or mask*) and (single or double or triple or treble)).tw.

13 crossover procedure/

14 double blind procedure/ or single blind procedure/

15 randomization/ or placebo/

16 parallel design/ or Latin square design/

17 randomized controlled trial/

18 controlled clinical trial/

19 10 or 12 or 13 or 14 or 15 or 16 or 17 or 18

20 11 and 19

21 (bovine or cattle or livestock).m_titl.

22 20 not 21

Cochrane Central Register of Controlled Trials

Issue 2 of 12, February 2023

#1 Tuberculosis

#2 Rifampin or rifampicin or ethambutol or Ethambutol or isoniazid or pyrazinamide

#3 #1 or #2

**Appendix 3: Details of data extraction form**

The data extraction form included:

*Source*: lead author, year of publication, journal, PubMed ID, sponsor, funding source, trial registration number, corresponding author

*Methods*: study design, study duration, sequence generation, allocation concealment, blinding, completeness of outcome data, selective reporting, other concerns about bias

*Participants:* number recruited, country/countries, patient type (new/retreatment), setting, inclusion/exclusion criteria, age, sex, HIV status, primary prophylaxis and availability of antiretroviral treatment, diabetes mellitus status, nutritional status

*Microbiological methods:* details of diagnostic and susceptibility testing methods

*Pharmacology:* availability of pharmacokinetic and/or pharmacogenetic data

*Interventions:* number of arms, names of drugs, doses of drugs, frequency of dosing, use of fixed dose combinations, duration and structure of regimen, use of direct observation

*Outcomes:* for each review outcome, where available, we extracted the number randomised in each arm, the number included in the analysis and the number experiencing the event to enable intention to treat (ITT), per protocol (PP) and complete case analysis.

**Appendix 4: Definitions used for primary and secondary outcomes in protocol**

Definitions consistent with WHO programmatic outcomes (Definitions and reporting framework for tuberculosis – 2013 revision: updated December 2014 and January 2020. <https://www.who.int/publications/i/item/9789241505345>: World Health Organisation; 2020. Licence: CC BY-NC-SA 3.0 IGO) were used for outcome measures but adapted to permit evaluation of regimens of variable length.

Primary outcomes

*Treatment success*

In adults with pulmonary TB (bacteriologically confirmed, presumed drug-sensitive): smear or culture negative in the last month of treatment and on at least one previous occasion OR completed treatment without evidence of failure but with no record to show that sputum smear or culture results in the last month of treatment and on at least one previous occasion were negative, either because the tests were not done or because results were unavailable.

In adults with extrapulmonary TB: resolution of clinical signs and symptoms of TB at the end of treatment as defined by the investigators.

*Treatment failure*

In adults with pulmonary TB: sputum smear or culture positive within the last month of treatment.

In adults with extrapulmonary TB: failure to resolve or return of clinical signs and symptoms of TB by the end of treatment as judged by the investigators.

*Relapse*

In adults with pulmonary TB: smear or culture positive on one or more occasions during a defined period of follow-up after having previous met the criteria for treatment success.

In adults with extrapulmonary TB: return of clinical signs and symptoms of TB as defined by the investigators after having previously met the criteria for treatment success.

*Death*

Death from any cause before starting or during treatment or follow-up.

*Adverse events*

All reported adverse events expressed as a number of participants experiencing an event.

Secondary outcomes

*Serious adverse events*

All adverse events detailed in the study report that either a) result in death, b) are life-threatening, c) require hospitalisation or prolongation of existing hospitalisation, d) result in persistent or significant disability or incapacity, e) consist of a congenital anomaly or birth defect or f) are another important (protocol-defined) medical condition.

*Drug-specific adverse events of interest*

Drug-induced liver injury or rifamycin hypersensitivity syndromes (thrombocytopaenia, influenza-like illness).

*Disease-specific efficacy outcomes of interest*

Outcomes specific to trials recruiting people with extrapulmonary forms of TB for example neurological disability in meningeal TB and pericardial constriction in pericardial TB.

**Appendix 5: Table of excluded studies**

| **Study** | **Reason for exclusion** |  |
| --- | --- | --- |
| Said2021 | PD analysis of already included study (Aarnoutse2017) |  |
| Clinicaltrials.gov  NCT05383742 | Ongoing study not yet reported |  |
| Clinicaltrials.gov  NCT04694586 | Suspended study not yet reported |  |
| Thanyanuch2021 | Wrong intervention |  |
| Clinicaltrials.gov  NCT05575518 | Ongoing study not yet reported |  |
| Espinosa-Pereiro2022 | Protocol only |  |
| Ekqvist2022 | Protocol only |  |
| VanWijk2023 | Linezolid pharmacokinetics poster from ongoing study not yet reported |  |
| Clinicaltrials.gov  NCT04738812 | Ongoing study not yet reported |  |
| Clinicaltrials.gov  NCT04951986 | Ongoing study not yet reported |  |
| Kwak2022 | Protocol only |  |
| Kengo2022 | Conference abstract of included study (Sekaggya-Wiltshire2023) |  |
| Kengo2023 | PK paper for included study (Sekaggya-Wiltshire2023) |  |
| Maitre2022 | Protocol only |  |
| Upton2023 | Abstract only, insufficient information provided | |
| Ngo2024 | Wrong comparator (flat dose RIF) | |
| Inbaraj2024 | Protocol only | |
| Trialsearch.who.int  CTRI/2023/05/053314 | Same study as Inbaraj2024 | |
| Clinicaltrials.gov  NCT05917340 | Same study as Inbaraj2024 | |
| Clinicaltrials.gov  NCT06498414 | Wrong intervention | |
| Clinicaltrials.gov  NCT06057519 | Ongoing study not yet reported | |
| Feng2023 | Wrong intervention | |
| Trialsearch.who.int  PACTR202306908320967 | Ongoing study not yet reported | |
| Ruslami2024 | Wrong intervention | |
| Paton2023 | Conference abstract of included study (Paton2023) | |
| Papineni2023 | Conference abstract of included study (Paton2023) | |
| Namale2024 | Protocol only | |
| Kibengo2023 | Conference abstract of ongoing study | |
| Souleymane2023^18^ | Wrong population (adults and children and no evidence as to split) | |

Said BN, Heysell SK, Yimer G, Aarnoutse RE, Kibiki GS, Mpagama S et al. Pharmacodynamic biomarkers for quantifying the mycobacterial effect of high doses of rifampin in patients with rifampin-susceptible pulmonary tuberculosis. *Int J Mycobacteriol*. 2021;10(4):457-62

Thanyanuch A, Charoen C, Surakameth M, Sukanya W. NAT2 Genotype-guided INH dosage to reduce drug-induced liver injury in Thai patients. *Sys Rev Pharm*. 2021;12(1):642-6

Espinosa-Pereiro J, Ghimire S, Sturkenboom MGG, Alffenaar J-WC, Tavares N, Aguirre S et al. Safety of rifampicin at high dose for difficult-to-treat tuberculosis: protocol for RIAlta Phase 2b/c trial. *Pharmaceutics*. 2023;15(1):9

Ekqvist D, Bornefall A, Augustinsson D, Sonnerbrandt M, Nordvall MJ, Fredrikson M et al. Safety and pharmacokinetics-pharamacodynamics of a shorter tuberculosis treatment with high-dose pyrazinamide and rifampicin: a study protocol of a phase II clinical trial (HighShort-RP). *BMJ Open*. 2022;12(3):e054788

Van Wijk R, Kibengo F, Kafeero P, Kabarambi A, Muhumuza P, Nakimbugwe M et al. Linezolid reaches target site in tuberculous meningitis patients irrespective of standard of high rifampin dose. Poster session presented at: American Society for Clinical Pharmacology and Therapeutics. Conference date range 2023 MAR 21; Atlanta, USA

Kwak N, Jeon D, Park Y, Kang YA, Kim KJ, Kim YR et al. Treatment shortening of drug-sensitive pulmonary tuberculosis using high-dose rifampicin for 3 months after culture conversion (Hi-DoRi-3): a study protocol for an open-label randomized clinical trial. *Trials*. 2022;23(1):666

Kengo A, Nabisere R, Gausi K, Musaazi J, Buzibye A, Aarnoutse R et al. Dolutegravir pharmacokinetics in patients receiving standard and higher doses of rifampicin. Conference abstract presented at: World Conference on Pharmacometrics. Conference date range 2022 MAR 29-APR 01; Cape Town, South Africa

Kengo A, Nabisere R, Gausi K, Musaazi J, Buzibye A, Omali D et al. Dolutegravir pharmacokinetics in Ugandan patients with TB and HIV receiving standard- versus high-dose rifampicin. *Antimicrob Agents Chemother*. 2023;67(11):e0043023

Upton C, de Jager V, Simonsson U, Mockeliunas L, Mabuka T, Lelievre J et al. Early bactericidal activity of meropenem, ertapenem, amoxicillin/clavulanate and optimised rifampicin in pulmonary tuberculosis. Oral abstract presented at: The Eleventh EDCTP Forum. Conference date range 2023 NOV 07-10; Paris, France

Ngo HX, Xu A, Velasquez GE, Zhang N, Chang VK, Kurbatova EV et al. Pharmacokinetic-pharmacodynamic evidence from a phase 3 trial to support flat-dosing of rifampicin for tuberculosis. *Clin Infect Dis*. 2024;78(6):1680-89

Feng Z, Miao Y, Peng Y, Sun F, Zhang Y, Li R et al. Optimizing (O) rifapentine-based (RI) regimen and shortening (EN) the treatment of drug-susceptible tuberculosis (T) (ORIENT) using an adaptive seamless design: study protocol of a multicenter randomized controlled trial. *BMC Infect Dis*. 2023;23(1):300

Ruslami R, Fregonese F, Apriani L, Barss L, Bedingfield N, Chiang V et al. High-dose, short-duration versus standard rifampicin for tuberculosis preventive treatment: a partially blinded, three-arm, non-inferiority, randomised, controlled trial. *Lancet Respir Med*. 2024;12(6):433-43

Paton N, Cousins C, Lu Q, Moorakonda R, Suresh C, Burhan E et al. Efficacy and safety of 8-wk tuberculosis treatment regimens in the Truncate-Tb trial. Oral abstract presented at: CROI. Conference date range 2023 FEB 19-22; Seattle, USA

Inbaraj LR, Manesh A, Ponnuraja C, Bhaskar A, Srinivasalu VA, Daniel BD. Comparative evaluation of intensified short course regimen and standard regimen for adults TB meningitis: a protocol for an open label, multi-center, parallel arms, randomized controlled superiority trial (INSHORT trial). Trials. 2024;25(1):294

Namale PE, Boloko L, Vermeulen M, Haigh KA, Bagula F, Maseko A et al. Testing novel strategies for patients hospitalised with HIV-associated disseminated tuberculosis (NewStrat-TB): protocol for a randomised controlled trial. *Trials*. 2024;25(1):311

Papineni P, Cousins C, Suresh C, Paton N. Results from TRUNCATE-TB-time for a change in strategy for TB-treatment shortening?. Conference abstract presented at: 25^th^ meeting of the British Infection Association. Conference date range 2023 MAY 16-17; Manchester, UK

Kibengo F, Kabarambi A, Kafeero P, Muhumuza P, Nakimbugwe M, Ssemaganda A et al. Safety and tolerability of adjunctive linezolid with high or standard dose rifampin for the treatment of adults with HIV-associated tuberculous meningitis in Uganda: preliminary findings from the ALTER trial. Conference abstract presented at: American Academy of Neurology Annual Meeting. Conference date range 2023 APR 22-27; Boston, USA

**Appendix 6: Definitions used in studies**

Definitions of treatment success used in included studies:

- culture conversion on LJ and mycobacterial growth indicator tube (MGIT) at 8 weeks (Aarnoutse2017, Atwine2020, Sekaggya-Wiltshire2023)
- culture conversion in liquid media within 12 weeks (Boeree2017)
- culture conversion on LJ media at 8 weeks (Jindani2016, Velasquez2018)
- culture conversion at 20 weeks (Long1979)
- sputum AFB smear and culture conversion 12 months after cure or treatment completion (Maug2020)
- not specified (oral presentation paper) (Merle2016)
- maintained culture negativity at minimum of 12 months (Jindani2023)

Definitions of treatment failure used in included studies:

- reporting failure but patients only followed up to twelve weeks (Aarnoutse2017)
- participant found to have multi-drug resistant TB on bone marrow examination six weeks into treatment (excluded therefore from this RIF sensitive TB review) (Atwine2020)
- participants judged to be clinically worse by study doctors, although the majority of these patients’ sputum cultures ‘remained negative’, or death from TB (Long1979)
- sputum smear AFB microscopy positive at month five (Maug2020)

**Appendix 7: Summary forest plots to graphically display findings from meta-analyses**

Analysis 1. Treatment success intention to treat analysis of risk ratio comparing rifampicin doses of 10mg/kg vs 15mg/kg


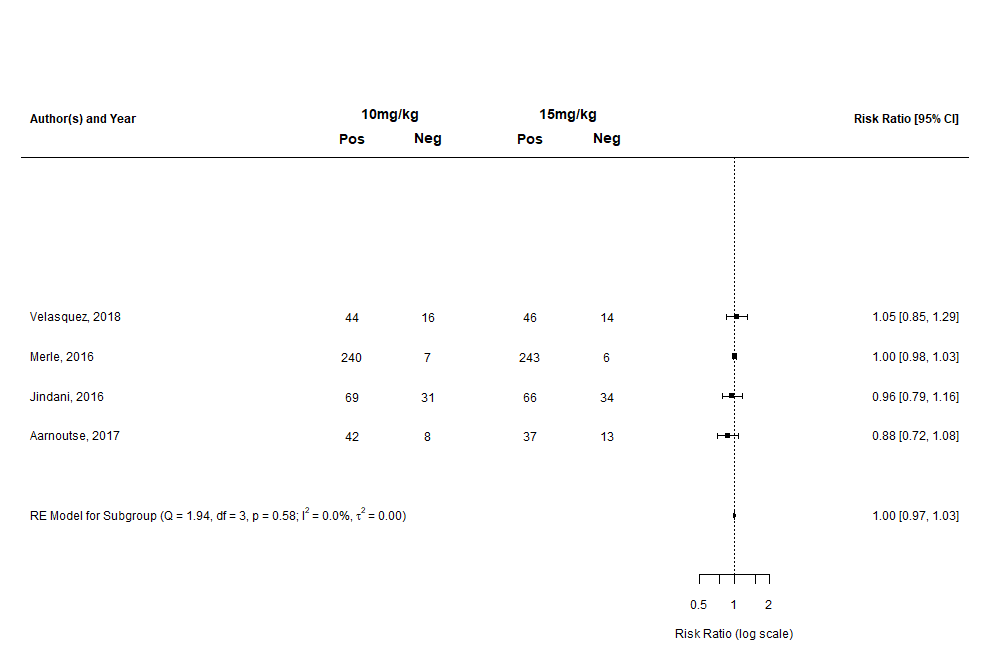


Analysis 2. Treatment success intention to treat analysis of risk ratio comparing rifampicin doses of 10mg/kg vs 20mg/kg and 10mg/kg vs 23mg/kg


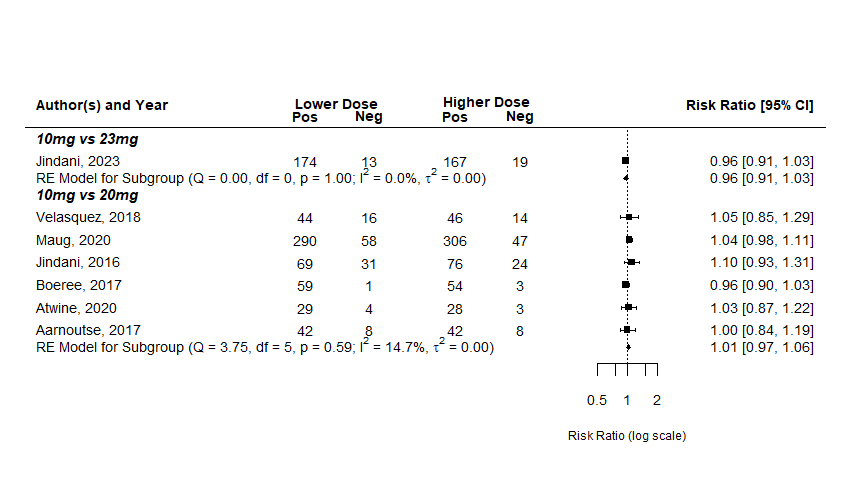


Analysis 3. Treatment success intention to treat analysis of risk ratio comparing rifampicin doses of 10mg/kg vs 35mg/kg


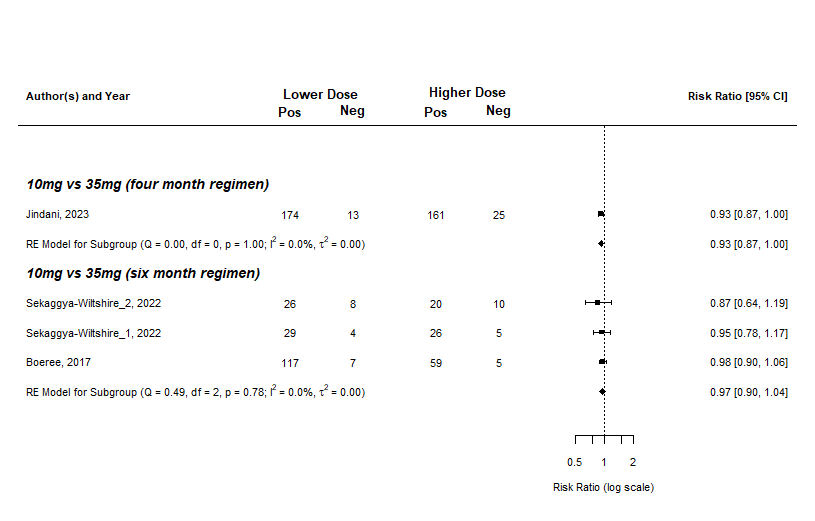
Analysis 4. Treatment success per protocol analysis of risk ratio comparing rifampicin doses of 10mg/kg vs 15mg/kg and 10mg/kg vs 20mg/kg


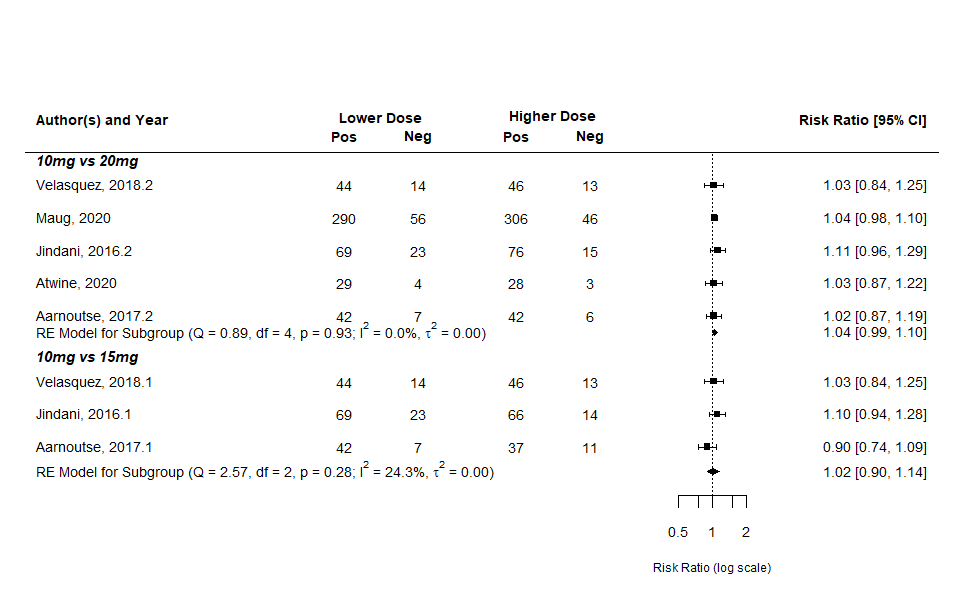


*Treatment success data from Boeree2017 was included in ITT but not PP analysis as the number of participants reported as having culture converted in one group (RIFQHZ) was higher than the number of participants included in total in their published mITT analysis (table 2 in report^13^). Treatment success data from Merle2016^32^ was also included in ITT but not PP analysis as the results were only available as an oral presentation with PP denominator values not provided.*

Analysis 5. Treatment failure intention to treat analysis of risk ratio comparing rifampicin doses of 10mg/kg vs 15mg/kg, 10mg/kg vs 20mg/kg and 10mg/kg vs 23mg/kg


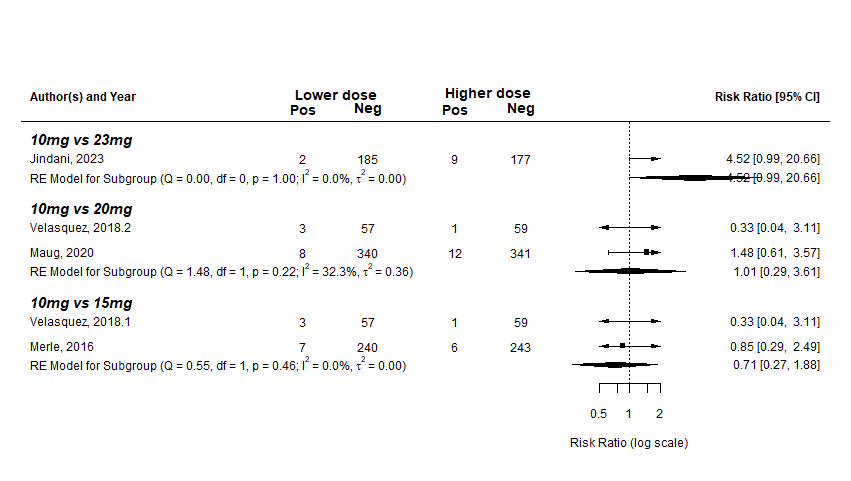


*Exact same numbers for Jindani2023 35mg/kg arm as above 23mg/kg arm*

Analysis 6. Relapse intention to treat analysis of risk ratio comparing rifampicin doses of 10mg/kg vs 15mg/kg and 10mg/kg vs 20mg/kg


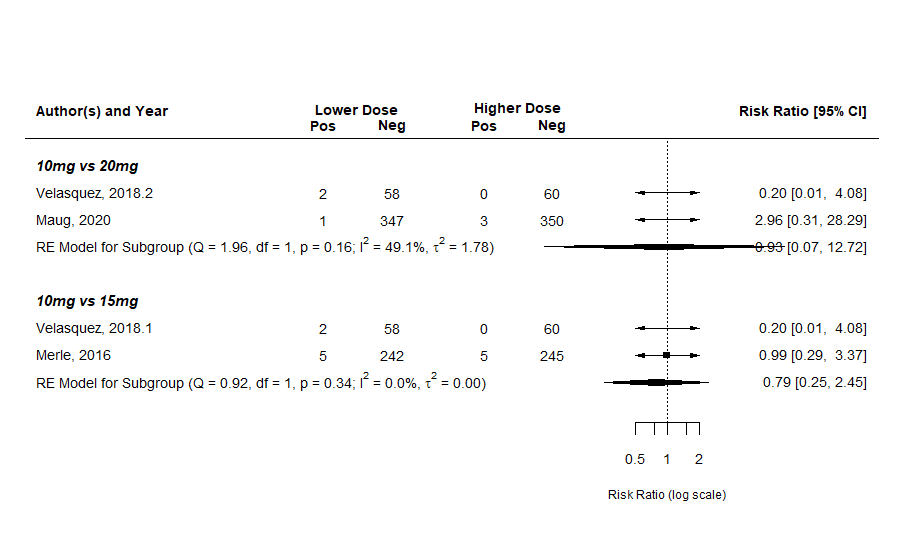


*Relapse data were not able to be reliably extracted from Long1979 as denominator values were inconsistent^38^*

Analysis 7. Relapse intention to treat analysis of risk ratio comparing rifampicin doses of 10mg/kg vs 23mg/kg and 10mg/kg vs 35mg/kg


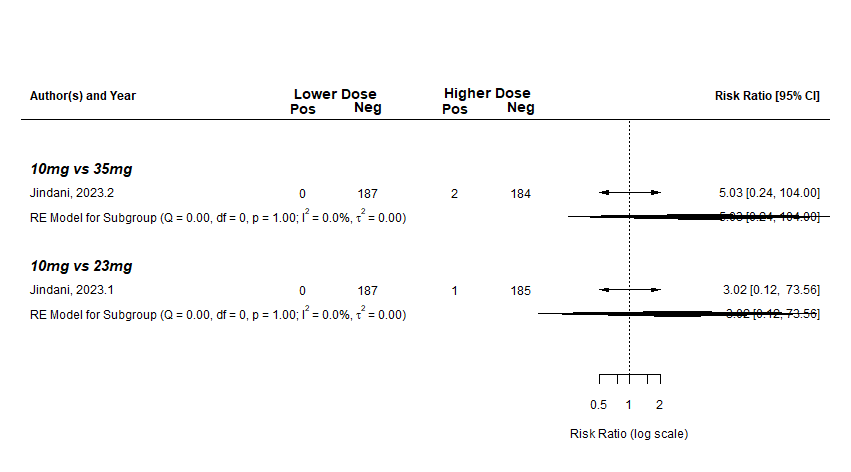


Analysis 8. All-cause mortality intention to treat analysis of risk ratio comparing rifampicin doses of 10mg/kg vs 15mg/kg


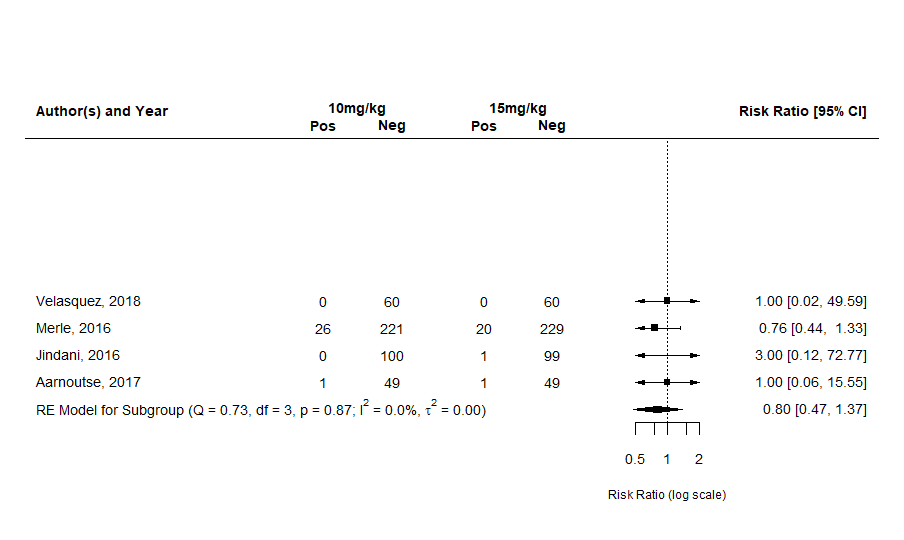


Analysis 9. All-cause mortality intention to treat analysis of risk ratio comparing rifampicin doses of 10mg/kg vs 20mg/kg and 10mg/kg vs 25mg/kg


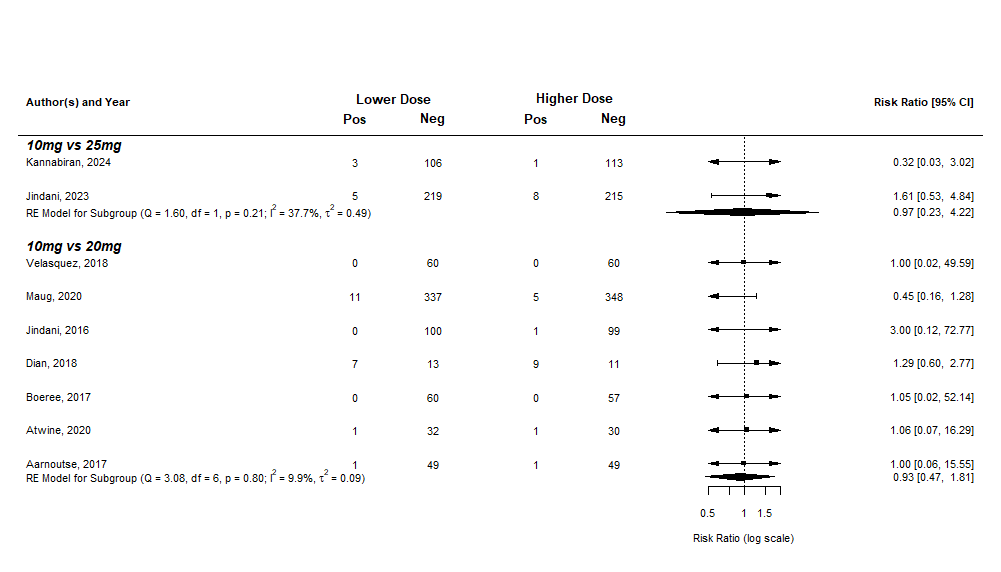


Analysis 10. All-cause mortality intention to treat analysis of risk ratio comparing rifampicin doses of 10mg/kg vs 30mg/kg and 10mg/kg vs 35mg/kg


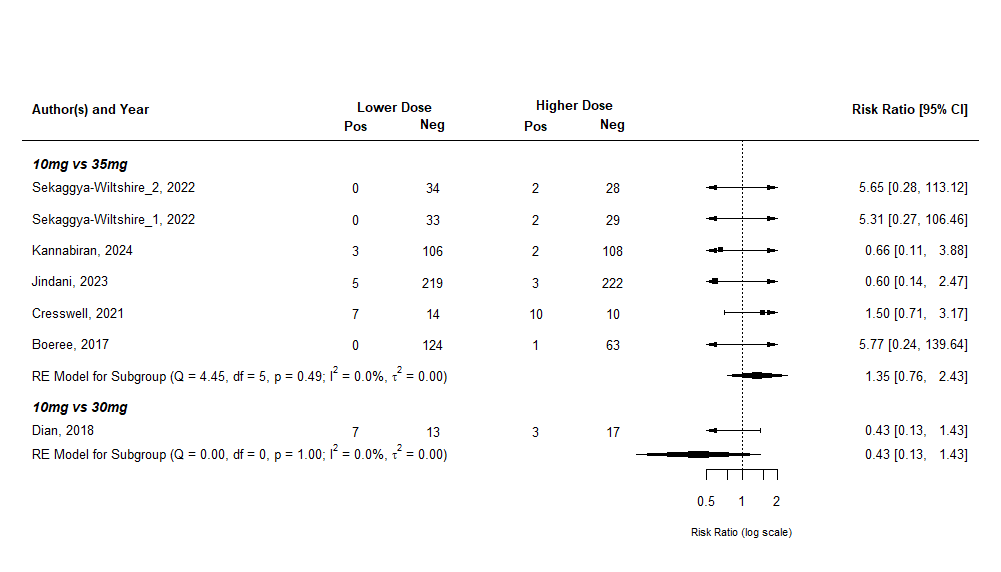


Analysis 11. All-cause mortality per protocol analysis of risk ratio comparing rifampicin doses of 10mg/kg vs 15mg/kg and 10mg/kg vs 20mg/kg


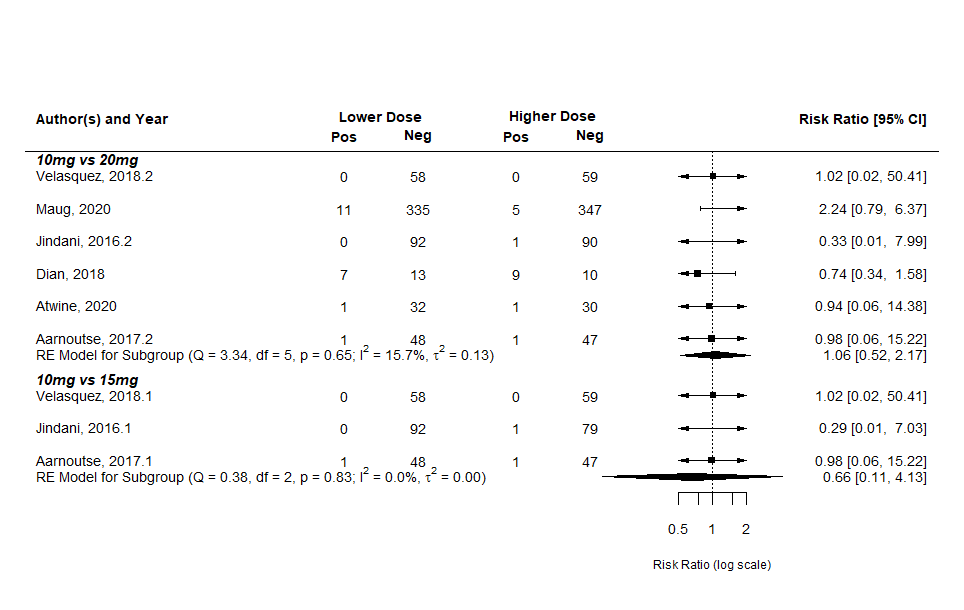


Analysis 12. TB-related mortality intention to treat analysis of risk ratio comparing rifampicin doses of 10mg/kg vs 25mg/kg and 10mg/kg vs 35mg/kg


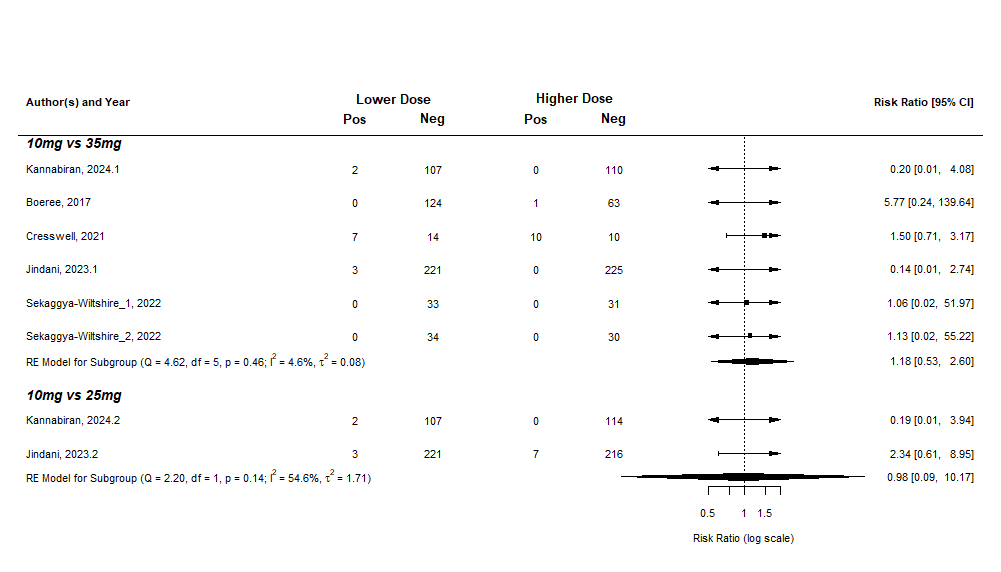


Analysis 13. Serious adverse events intention to treat analysis of risk ratio comparing rifampicin doses of 10mg/kg vs 15mg/kg and 10mg/kg vs 20mg/kg


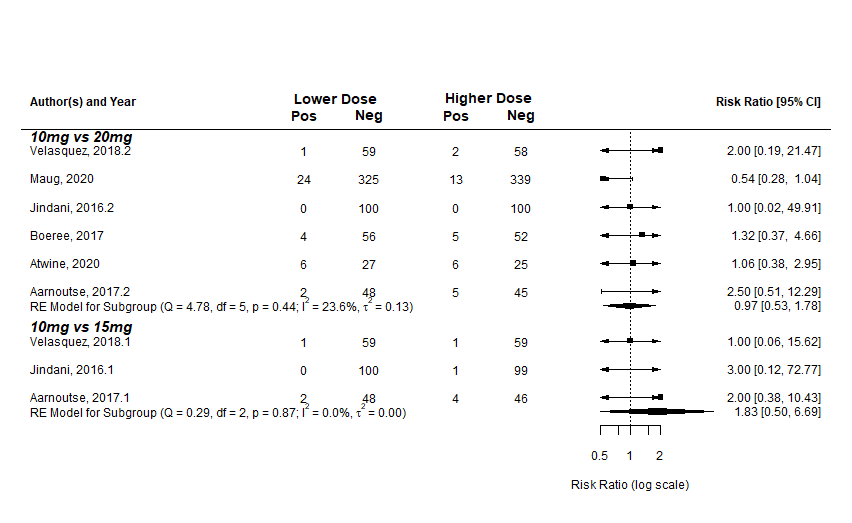


Analysis 14. Serious adverse events intention to treat analysis of risk ratio comparing rifampicin doses of 10mg/kg vs 25mg/kg and 10mg/kg vs 35mg/kg


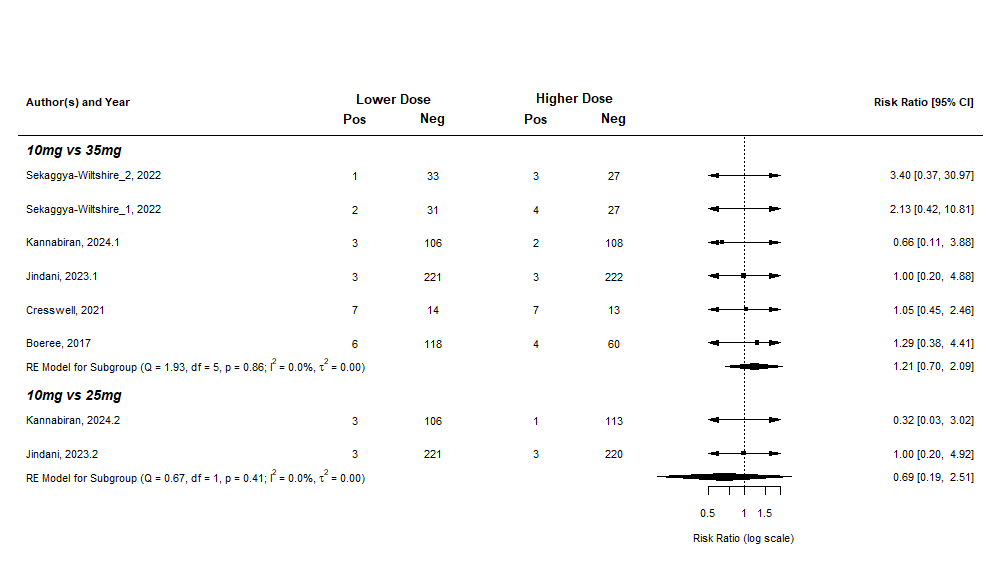


Analysis 15. Drug-induced liver injury intention to treat analysis of risk ratio comparing rifampicin 10mg/kg vs 15mg/kg


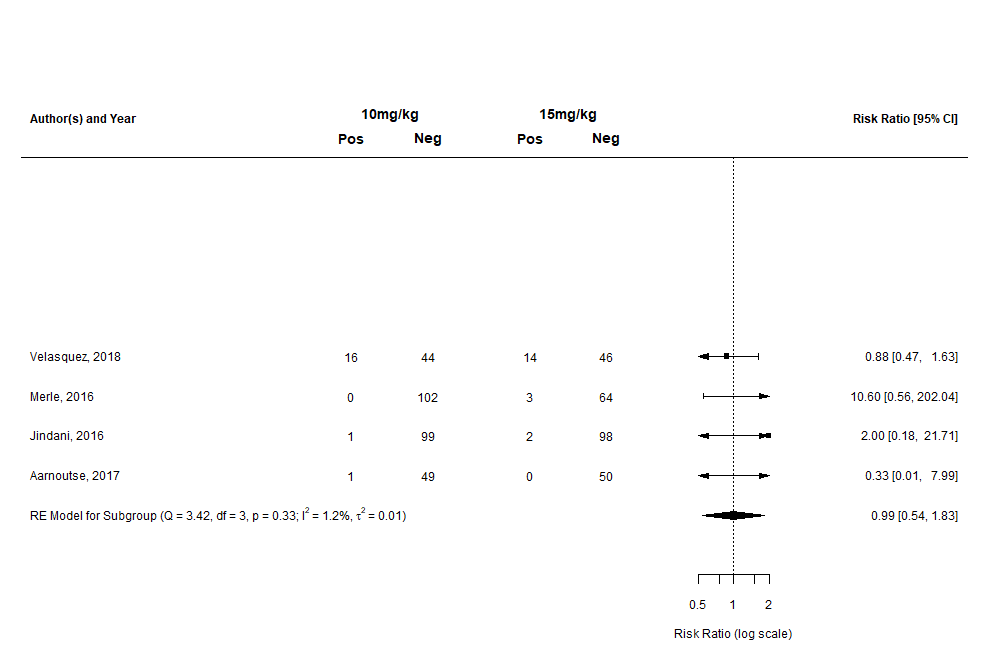


Analysis 16. Drug-induced liver injury intention to treat analysis of risk ratio comparing 10mg/kg vs 20mg/kg and 10mg/kg vs 25mg/kg


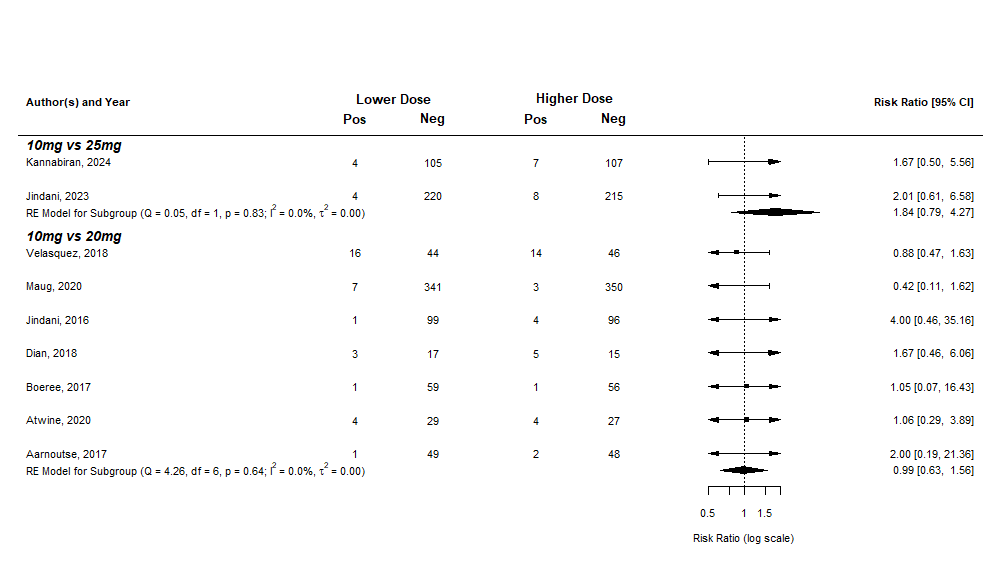


Analysis 17. Drug-induced liver injury intention to treat analysis of risk ratio comparing 10mg/kg vs 30mg/kg and 10mg/kg vs 35mg/kg


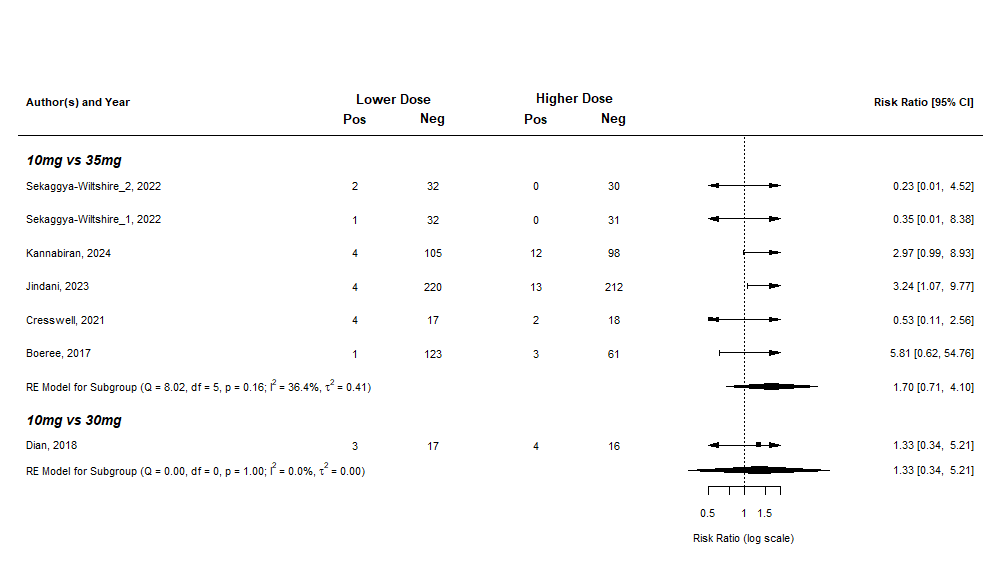


**Appendix 8: Data from studies not included in meta-analyses owing to non-comparable background regimens**

Intention to treat risk ratios comparing standard 10mg/kg doses of rifampicin with higher doses with varying background regimens.

| **Paper** | **Dose comparison** | **Additional intervention** | **Outcome** | **Existing cumulative/**  **individual** | **Stats, ITT (RR [95% confidence intervals])** |
| --- | --- | --- | --- | --- | --- |
| Atwine  2020^30^ | 10vs20 | Nil | Tx success | Cumulative | 1.01 [0.97-1.06] |
|  |  | Different dose of EFV |  | Individual | 1.07 [0.92-1.25] |
|  |  | Nil | Mortality | Cumulative | 0.93 [0.47-1.81] |
|  |  | Different dose of EFV |  | Individual | 1.00 [0.07-15.33] |
| Boeree  2017^13^ | 10vs20 | Nil | Tx success | Cumulative | 1.01 [0.97-1.06] |
|  |  | Moxifloxacin |  | Individual | 0.99 [0.92-1.07] |
|  |  | Nil | Mortality | Cumulative | 0.93 [0.47-1.81] |
|  |  | Moxifloxacin |  | Individual | 1.95 [0.04-97.30] |
| *Note on Boeree that original comparison both 10 and 20 groups had additional SQ109; these data use the standard RHZE control* | | | | | |
| Heemskerk  2016^15^ | 10vs15 | Nil | Mortality | Cumulative | 0.80 [0.47-1.37] |
|  |  | Levofloxacin |  | Individual | 0.99 [0.80-1.24] |
| Davis  2023^26^ | 10vs35/20 | Nil | Mortality | Cumulative | 1.48 [0.80-2.75] |
|  |  | Linezolid |  | Individual | 0.89 [0.17-4.67] |
|  |  | Linezolid and aspirin |  | Individual | 1.25 [0.29-5.38] |
| *Note on Davis that high dose arm was either IV20 or PO35, so not directly comparable although cumulative 10vs35 data presented here* | | | | | |
| Paton  2023^16^ | 10vs35/20 | Nil | Mortality | Cumulative | 1.48 [0.80-2.75] |
|  |  | Linezolid |  | Individual | 1.64 [0.40-6.76] |
|  |  | Clofazimine |  | Individual | 0.33 [0.02-6.30] |
| *Note on Paton that high dose arm was 10vs35 then changed to 10vs20, so not directly comparable although cumulative 10vs35 data presented here* | | | | | |

**Appendix 9: Reporting on other secondary outcomes**

*Discontinuation*

Discontinuation was not consistently reported.

*Thrombocytopaenia*

Thrombocytopaenia data were reported in eight studies^14,15,26,27,29,30,34,38^. Data were not directly comparable; all reported very low or no events and found no significant difference between arms.

*Rifamycin hypersensitivity*

Rifamycin hypersensitivity was discussed in six studies^8,14,27,29,36,38^. One study reported episodes of hypersensitivity, two were mild and one was an anaphylactic reaction following administration of both intravenous RIF and moxifloxacin; which agent had caused the anaphylaxis was unknown^14^. Studies reporting on hypersensitivity included 1,323 participants, suggesting a crude rate of <0.003% and no interpretable relationship with dose.

*Disease-specific efficacy outcomes*

Disease-specific efficacy outcomes were not consistently reported. The six studies of participants with tuberculous meningitis variably reported neurological outcomes, but follow-up was frequently short and study reports placed more emphasis on mortality data.

**Appendix 10: Risk of bias tables for efficacy and safety outcomes; notes on bias interpretation**

Figure i: risk of bias for efficacy outcomes


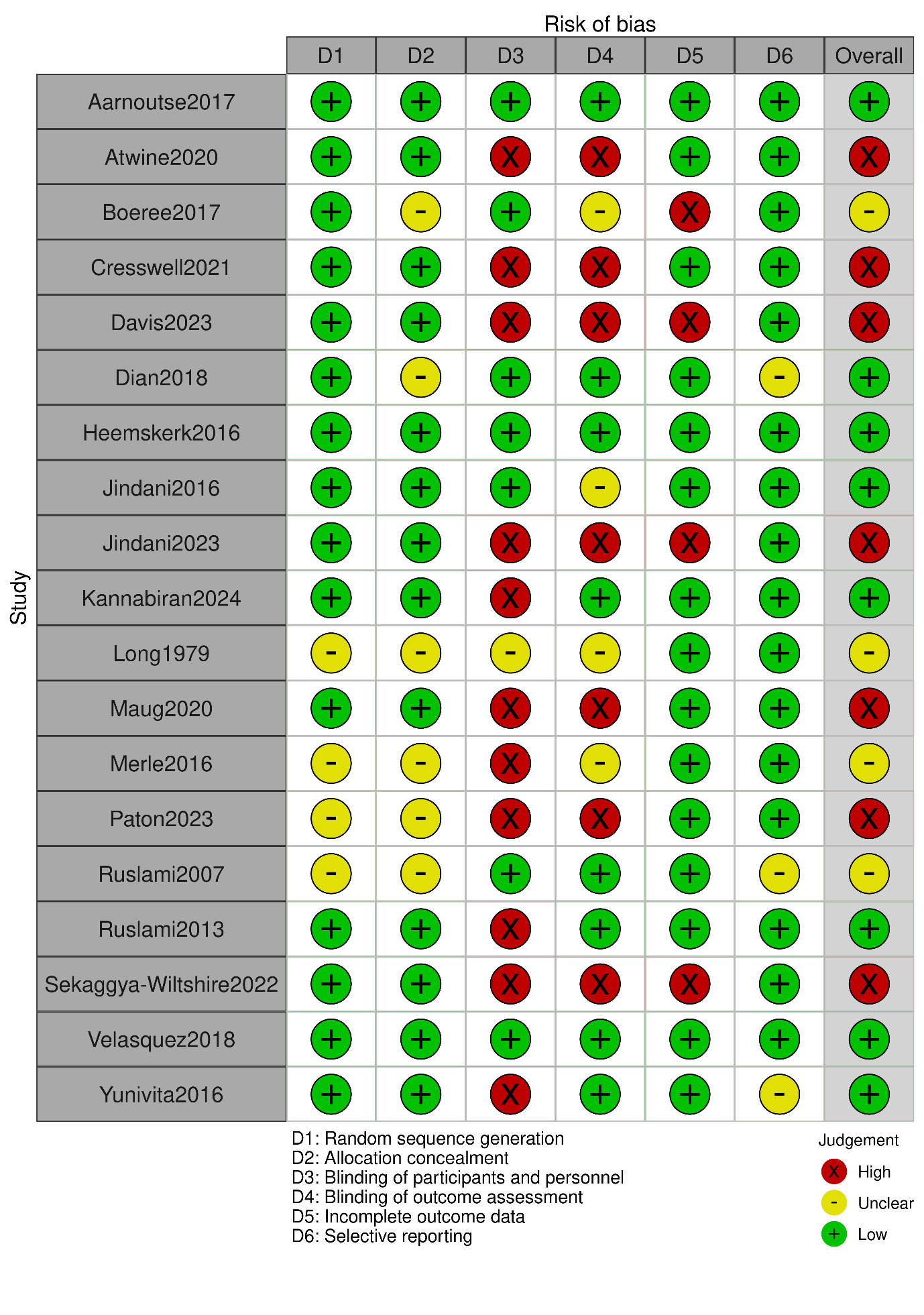


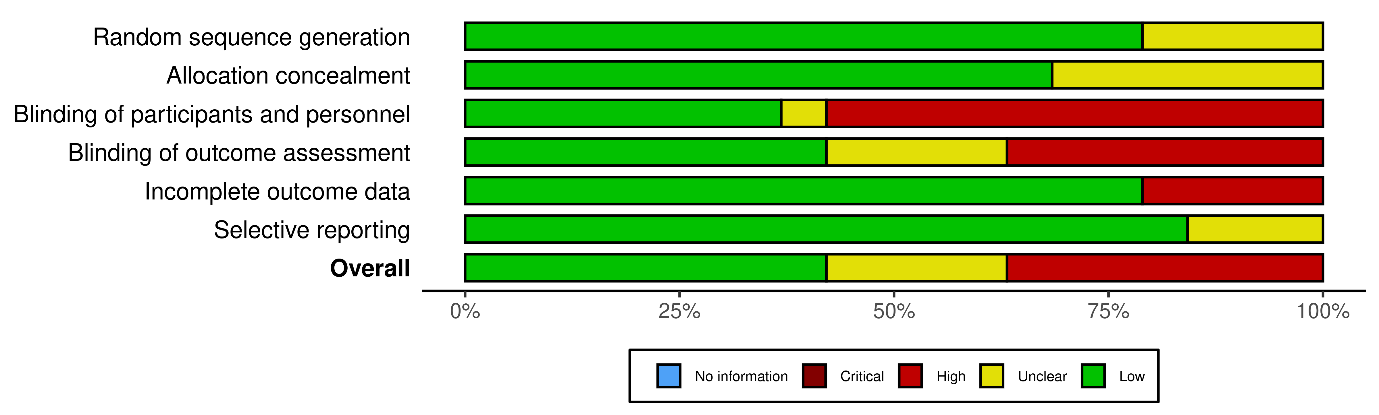


Figure ii: risk of bias for safety outcomes


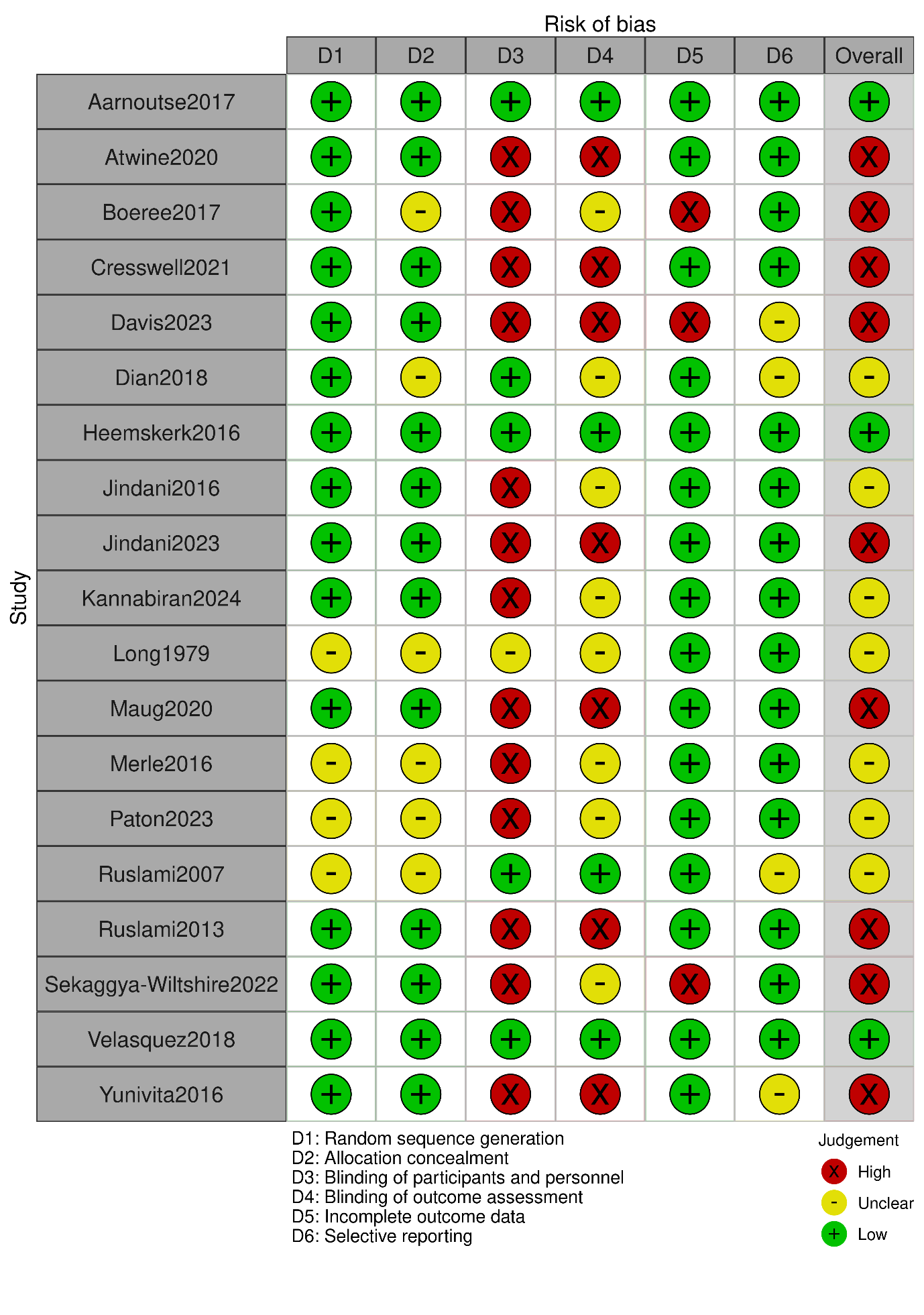


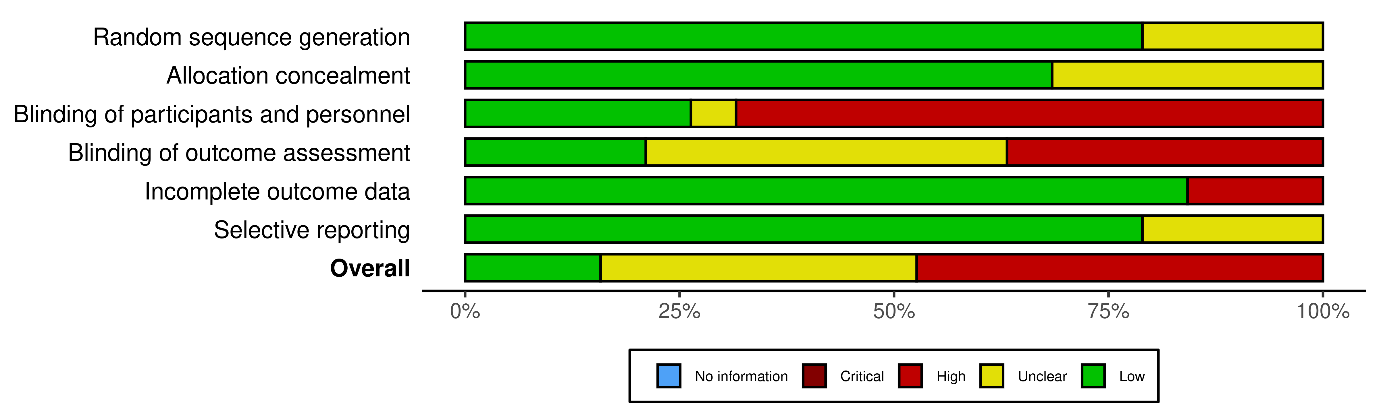


**Further details on bias interpretation and results**

Random sequence generation and allocation concealment were low risk of bias, where reported. Five studies reported double blinding procedures leading to low risk of performance bias. All but one of these studies reported concealing the treatment group to outcome assessors, leading to a low risk of detection bias. Thirteen studies described an open-label procedure, leading to a high risk of performance bias. Three open-label studies blinded their laboratory personnel, leading to an unclear risk of detection bias for safety outcomes and a low risk of bias for efficacy outcomes. The other fully open-label studies had a high risk of detection bias. In TBM studies where outcome data were predominantly mortality, blinding of outcome assessment would not impact efficacy data, resulting in a low risk of bias. All but three studies reported >90% follow-up of participants, leading to a generally low risk of attrition bias. The majority of studies had no evidence of selection bias, however, three studies did not report SAE data when outcomes related to safety, tolerability and/or adverse events, representing an unclear risk of selective reporting bias. Visual inspection of Funnel and Galbraith plots (appendix 12) produced for meta-analyses of primary outcomes incorporating three or more trials did not reveal any clear evidence of publication bias.

Participants were randomised in error in a few studies^13,16,26,28^. Two studies changed inclusion criteria during the trial; one removed a lower weight and CD4 count limit^30^, the other changed to include participants with HIV with some stipulations^16^. One study reported an error whereby a weight band of participants received fewer fixed dose combination tablets than they should have been allocated^35^. One study changed the RIF mg/kg dose in intervention groups during the study^16^. All of these could lead to bias.

**Appendix 11: GRADE Summary of Findings table**

| **Certainty assessment** | | | | | | | **№ of patients** | | **Effect** | | **Certainty** | **Importance** |
| --- | --- | --- | --- | --- | --- | --- | --- | --- | --- | --- | --- | --- |
| **№ of studies** | **Study design** | **Risk of bias** | **Inconsistency** | **Indirectness** | **Imprecision** | **Other considerations** | **higher doses of rifampicin** | **standard doses of rifampicin** | **Relative (95% CI)** | **Absolute (95% CI)** |  |  |
| **Treatment success 10vs15mg/kg (ITT)** | | | | | | | | | | | | |
| 4 | randomised trials | not serious | not serious | not serious | not serious | none | 392/459 (85.4%) | 395/457 (86.4%) | **RR 1.00** (0.97 to 1.03) | **0 fewer per 1,000** (from 26 fewer to 26 more) | ⨁⨁⨁⨁ High |  |
| **Treatment success 10vs20mg/kg (ITT)** | | | | | | | | | | | | |
| 6 | randomised trials | not serious | not serious | not serious | not serious | none | 552/651 (84.8%) | 533/651 (81.9%) | **RR 1.01** (0.97 to 1.06) | **8 more per 1,000** (from 25 fewer to 49 more) | ⨁⨁⨁⨁ High |  |
| **Treatment success 10vs23mg/kg (ITT)** | | | | | | | | | | | | |
| 1 | randomised trials | not serious | not serious | not serious | serious^a^ | none | 167/186 (89.8%) | 174/187 (93.0%) | **RR 0.96** (0.91 to 1.03) | **37 fewer per 1,000** (from 84 fewer to 28 more) | ⨁⨁⨁◯ Moderate^a^ |  |
| **Treatment success 10vs35mg/kg (ITT, six month regimen)** | | | | | | | | | | | | |
| 2 | randomised trials | not serious | not serious | not serious | serious^b^ | none | 105/125 (84.0%) | 172/191 (90.1%) | **RR 0.97** (0.90 to 1.04) | **27 fewer per 1,000** (from 90 fewer to 36 more) | ⨁⨁⨁◯ Moderate^b^ |  |
| **Treatment success 10vs35mg/kg (ITT, four month regimen)** | | | | | | | | | | | | |
| 1 | randomised trials | not serious | not serious | not serious | serious^a^ | none | 161/186 (86.6%) | 174/187 (93.0%) | **RR 0.93** (0.87 to 1.00) | **65 fewer per 1,000** (from 121 fewer to 0 fewer) | ⨁⨁⨁◯ Moderate^a^ |  |
| **Treatment failure 10vs15mg/kg (ITT)** | | | | | | | | | | | | |
| 2 | randomised trials | not serious | not serious | not serious | very serious^b^ | none | 7/309 (2.3%) | 10/307 (3.3%) | **RR 0.71** (0.27 to 1.88) | **9 fewer per 1,000** (from 24 fewer to 29 more) | ⨁⨁◯◯ Low^b^ |  |
| **Treatment failure 10vs20mg/kg (ITT)** | | | | | | | | | | | | |
| 2 | randomised trials | not serious | not serious | not serious | very serious^b^ | none | 13/413 (3.1%) | 11/408 (2.7%) | **RR 1.01** (0.29 to 3.61) | **0 fewer per 1,000** (from 19 fewer to 70 more) | ⨁⨁◯◯ Low^b^ |  |
| **Treatment failure 10vs23mg/kg (ITT)** | | | | | | | | | | | | |
| 1 | randomised trials | not serious | not serious | not serious | very serious^a,b^ | none | 9/186 (4.8%) | 2/187 (1.1%) | **RR 4.52** (0.99 to 20.66) | **38 more per 1,000** (from 0 fewer to 210 more) | ⨁⨁◯◯ Low^a,b^ |  |
| **Treatment failure 10vs35mg/kg (ITT)** | | | | | | | | | | | | |
| 1 | randomised trials | not serious | not serious | not serious | very serious^a,b^ | none | 9/186 (4.8%) | 2/187 (1.1%) | **RR 4.52** (0.99 to 20.66) | **38 more per 1,000** (from 0 fewer to 210 more) | ⨁⨁◯◯ Low^a,b^ |  |
| **Relapse 10vs15mg/kg (ITT)** | | | | | | | | | | | | |
| 2 | randomised trials | not serious | not serious | not serious | very serious^b^ | none | 5/310 (1.6%) | 7/307 (2.3%) | **RR 0.79** (0.25 to 2.45) | **5 fewer per 1,000** (from 17 fewer to 33 more) | ⨁⨁◯◯ Low^b^ |  |
| **Relapse 10vs20mg/kg (ITT)** | | | | | | | | | | | | |
| 2 | randomised trials | not serious | not serious | not serious | very serious^b^ | none | 3/413 (0.7%) | 3/408 (0.7%) | **RR 0.93** (0.07 to 12.72) | **1 fewer per 1,000** (from 7 fewer to 86 more) | ⨁⨁◯◯ Low^b^ |  |
| **Relapse 10vs23mg/kg (ITT)** | | | | | | | | | | | | |
| 1 | randomised trials | not serious | not serious | not serious | very serious^a,b^ | none | 1/186 (0.5%) | 0/187 (0.0%) | **RR 3.02** (0.12 to 73.56) | **0 fewer per 1,000** (from 0 fewer to 0 fewer) | ⨁⨁◯◯ Low^a,b^ |  |
| **Relapse 10vs35mg/kg (ITT)** | | | | | | | | | | | | |
| 1 | randomised trials | not serious | not serious | not serious | very serious^a,b^ | none | 2/186 (1.1%) | 0/187 (0.0%) | **RR 5.03** (0.24 to 104.00) | **0 fewer per 1,000** (from 0 fewer to 0 fewer) | ⨁⨁◯◯ Low^a,b^ |  |
| **All-cause mortality 10vs15mg/kg (ITT)** | | | | | | | | | | | | |
| 4 | randomised trials | not serious | not serious | not serious | serious^b^ | none | 22/459 (4.8%) | 27/457 (5.9%) | **RR 0.80** (0.47 to 1.37) | **12 fewer per 1,000** (from 31 fewer to 22 more) | ⨁⨁⨁◯ Moderate^b^ |  |
| **All-cause mortality 10vs20mg/kg (ITT)** | | | | | | | | | | | | |
| 7 | randomised trials | not serious | not serious | not serious | serious^b^ | none | 17/671 (2.5%) | 20/691 (2.9%) | **RR 0.93** (0.47 to 1.81) | **2 fewer per 1,000** (from 15 fewer to 23 more) | ⨁⨁⨁◯ Moderate^b^ |  |
| **All-cause mortality 10vs25mg/kg (ITT)** | | | | | | | | | | | | |
| 2 | randomised trials | not serious | not serious | not serious | serious^b^ | none | 9/337 (2.7%) | 5/224 (2.2%) | **RR 0.97** (0.23 to 4.22) | **1 fewer per 1,000** (from 17 fewer to 72 more) | ⨁⨁⨁◯ Moderate^b^ |  |
| **All-cause mortality 10vs30mg/kg (ITT)** | | | | | | | | | | | | |
| 1 | randomised trials | not serious | not serious | not serious | serious^a,b^ | none | 3/20 (15.0%) | 7/20 (35.0%) | **RR 0.43** (0.13 to 1.43) | **200 fewer per 1,000** (from 305 fewer to 150 more) | ⨁⨁⨁◯ Moderate^a,b^ |  |
| **All-cause mortality 10vs35mg/kg (ITT)** | | | | | | | | | | | | |
| 5 | randomised trials | not serious | not serious | not serious | serious^b^ | none | 20/480 (4.2%) | 14/545 (2.6%) | **RR 1.35** (0.76 to 2.43) | **9 more per 1,000** (from 6 fewer to 37 more) | ⨁⨁⨁◯ Moderate^b^ |  |
| **TB-related mortality 10vs25mg/kg (ITT)** | | | | | | | | | | | | |
| 2 | randomised trials | not serious | not serious | not serious | serious^b^ | none | 7/337 (2.1%) | 5/333 (1.5%) | **RR 0.98** (0.09 to 10.17) | **0 fewer per 1,000** (from 14 fewer to 138 more) | ⨁⨁⨁◯ Moderate^b^ |  |
| **TB-related mortality 10vs35mg/kg (ITT)** | | | | | | | | | | | | |
| 5 | randomised trials | not serious | not serious | not serious | serious^b^ | none | 11/480 (2.3%) | 12/545 (2.2%) | **RR 1.38** (0.70 to 2.73) | **8 more per 1,000** (from 7 fewer to 38 more) | ⨁⨁⨁◯ Moderate^b^ |  |
| **Serious adverse events 10vs15mg/kg (ITT)** | | | | | | | | | | | | |
| 3 | randomised trials | serious^c^ | not serious | not serious | serious^b^ | none | 6/210 (2.9%) | 3/210 (1.4%) | **RR 1.83** (0.50 to 6.69) | **12 more per 1,000** (from 7 fewer to 81 more) | ⨁⨁◯◯ Low^b,c^ |  |
| **Serious adverse events 10vs20mg/kg (ITT)** | | | | | | | | | | | | |
| 6 | randomised trials | serious^c^ | not serious | not serious | serious^b^ | none | 31/650 (4.8%) | 37/652 (5.7%) | **RR 0.97** (0.53 to 1.78) | **2 fewer per 1,000** (from 27 fewer to 44 more) | ⨁⨁◯◯ Low^b,c^ |  |
| **Serious adverse events 10vs25mg/kg (ITT)** | | | | | | | | | | | | |
| 2 | randomised trials | serious^c^ | not serious | not serious | serious^b^ | none | 4/337 (1.2%) | 6/333 (1.8%) | **RR 0.69** (0.19 to 2.51) | **6 fewer per 1,000** (from 15 fewer to 27 more) | ⨁⨁◯◯ Low^b,c^ |  |
| **Serious adverse events 10vs35mg/kg (ITT)** | | | | | | | | | | | | |
| 5 | randomised trials | serious^c^ | not serious | not serious | serious^b^ | none | 23/480 (4.8%) | 22/545 (4.0%) | **RR 1.29** (0.73 to 2.29) | **12 more per 1,000** (from 11 fewer to 52 more) | ⨁⨁◯◯ Low^b,c^ |  |
| **Drug-induced liver injury 10vs15mg/kg (ITT)** | | | | | | | | | | | | |
| 4 | randomised trials | serious^c^ | not serious | not serious | serious^b,d^ | none | 19/277 (6.9%) | 18/312 (5.8%) | **RR 0.99** (0.54 to 1.83) | **1 fewer per 1,000** (from 27 fewer to 48 more) | ⨁⨁◯◯ Low^b,c,d^ |  |
| **Drug-induced liver injury 10vs20mg/kg (ITT)** | | | | | | | | | | | | |
| 7 | randomised trials | serious^c^ | not serious | not serious | serious^b,d^ | none | 33/671 (4.9%) | 33/671 (4.9%) | **RR 0.99** (0.63 to 1.56) | **0 fewer per 1,000** (from 18 fewer to 28 more) | ⨁⨁◯◯ Low^b,c,d^ |  |
| **Drug-induced liver injury 10vs25mg/kg (ITT)** | | | | | | | | | | | | |
| 2 | randomised trials | serious^c^ | not serious | not serious | very serious^b^ | none | 15/337 (4.5%) | 8/333 (2.4%) | **RR 1.84** (0.79 to 4.27) | **20 more per 1,000** (from 5 fewer to 79 more) | ⨁⨁◯◯ Low^b,c^ |  |
| **Drug-induced liver injury 10vs30mg/kg (ITT)** | | | | | | | | | | | | |
| 1 | randomised trials | serious^c^ | not serious | not serious | very serious^a,b^ | none | 4/20 (20.0%) | 3/20 (15.0%) | **RR 1.33** (0.34 to 5.21) | **50 more per 1,000** (from 99 fewer to 632 more) | ⨁◯◯◯ Very low^a,b,c^ |  |
| **Drug-induced liver injury 10vs35mg/kg (ITT)** | | | | | | | | | | | | |
| 5 | randomised trials | serious^c^ | not serious | not serious | serious^b,d^ | none | 30/480 (6.3%) | 16/545 (2.9%) | **RR 1.70** (0.71 to 4.10) | **21 more per 1,000** (from 9 fewer to 91 more) | ⨁⨁◯◯ Low^b,c,d^ |  |

**CI:** confidence interval; **RR:** risk ratio

#### Explanations

a. Only one study included in this analysis

b. Outcomes within strata well below Optimal Information Size and low number of events

c. High risk of bias in 9 of the 18 included studies with regard to safety assessment owing to open-label methodology

d. Direction of effect varies significantly between studies

*Optimal Information Size: minimum amount of information required in the literature to reach reliable conclusions about an intervention.*

**Appendix 12: Funnel and Galbraith plots to assess for publication bias**

Treatment success

10mg/kg versus 15mg/kg


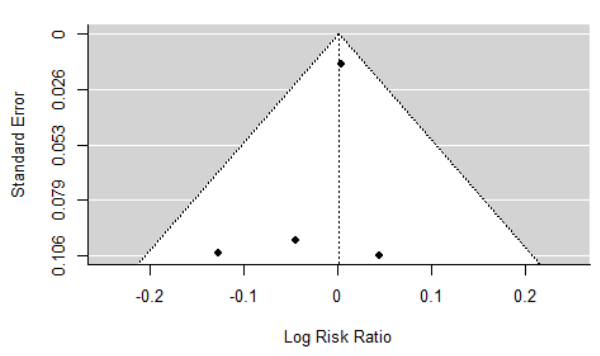

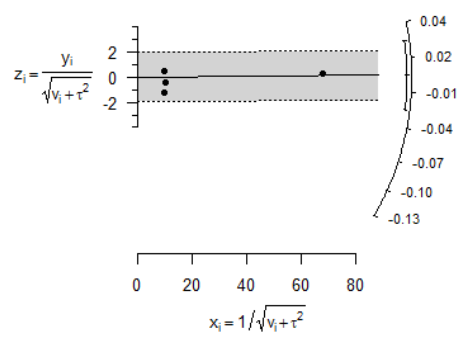


10mg/kg versus 20mg/kg


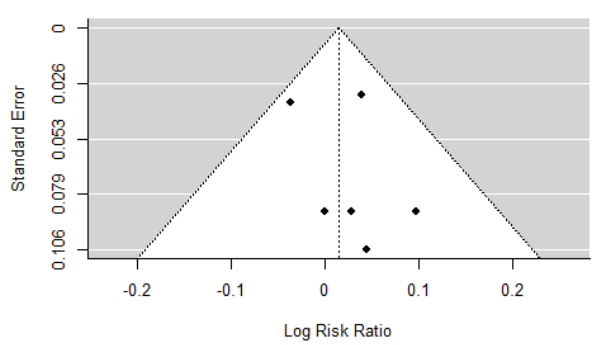

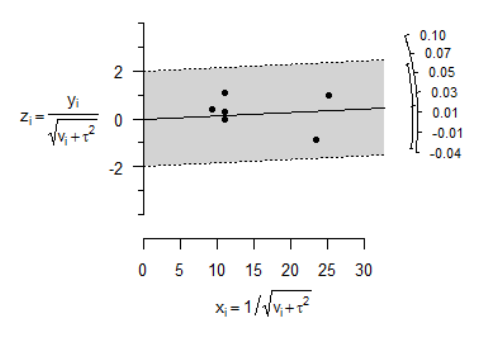


All-cause mortality

10mg/kg versus 15mg/kg


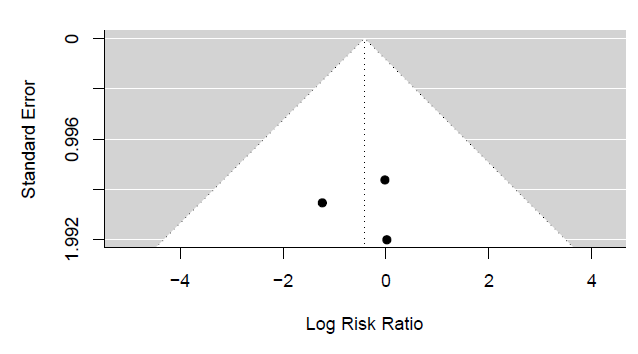

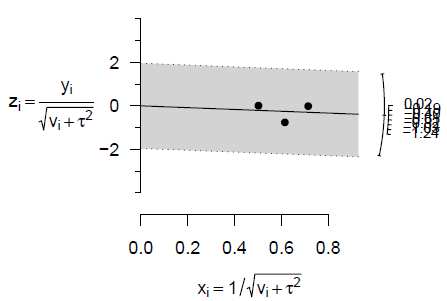


10mg/kg versus 20mg/kg


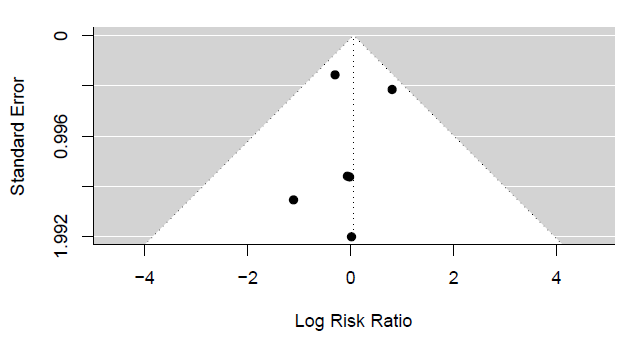

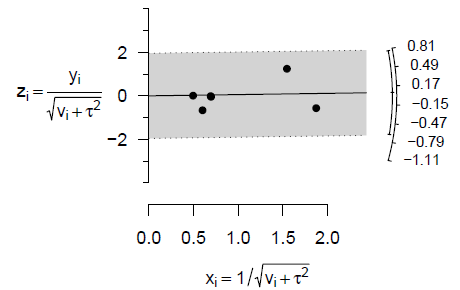


10mg/kg versus 35mg/kg


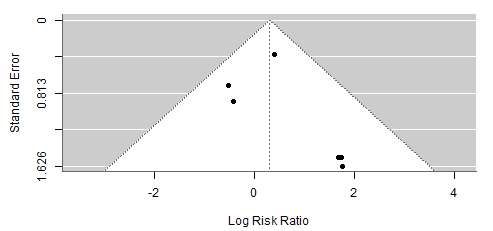

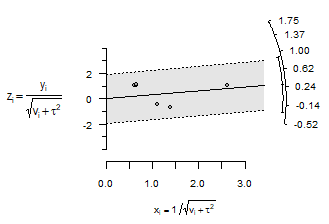


Serious adverse events

10mg/kg versus 15mg/kg


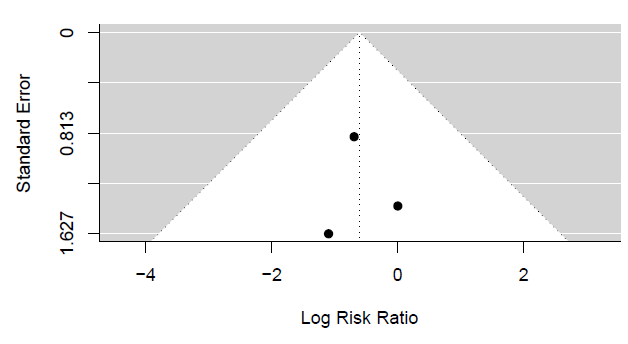

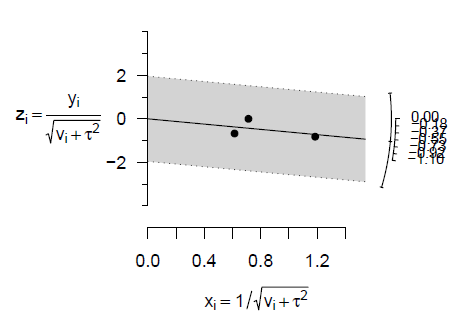


10mg/kg versus 20mg/kg


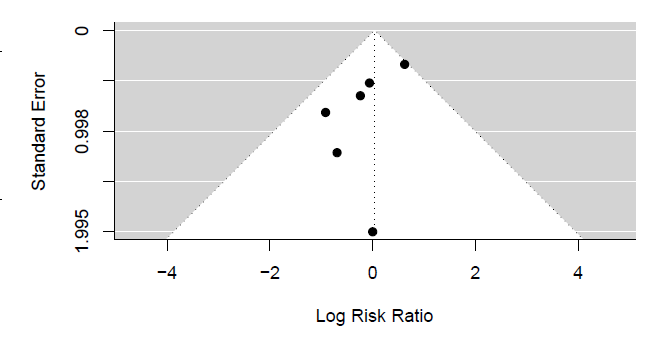

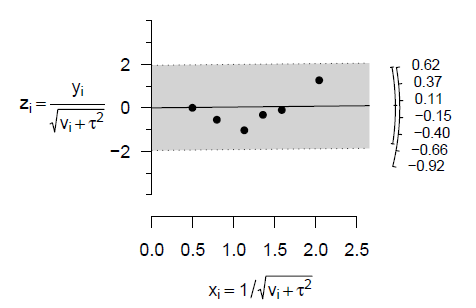


10mg/kg versus 35mg/kg


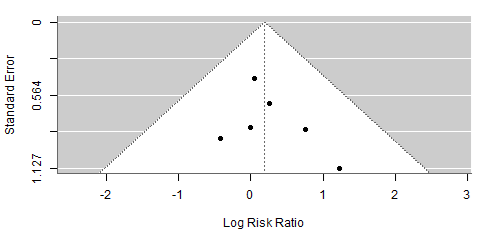

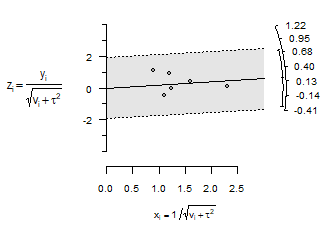


Drug-induced liver injury

10mg/kg vs 15mg/kg


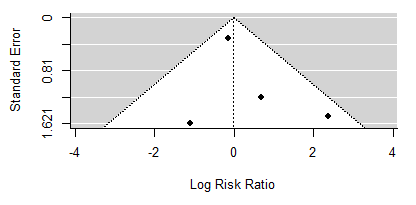

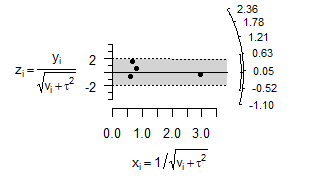


10mg/kg vs 20mg/kg


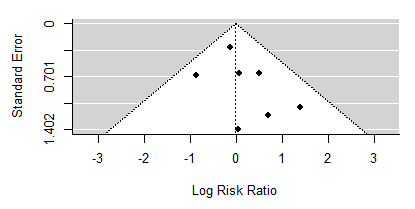

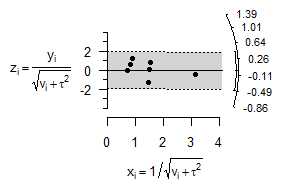


10mg/kg vs 35mg/kg


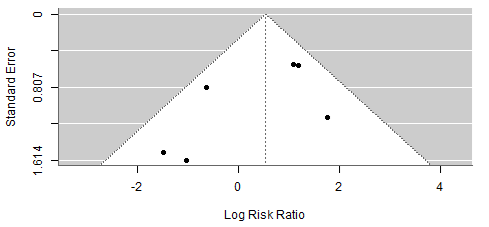

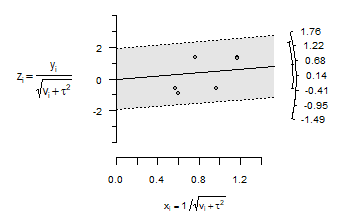

Supplement: Appendices 1–12 [file mmc1.docx]
